# Supplementary material for: Large-Scale Comparative Analysis of Codon Models Accounting for Protein and Nucleotide Selection
Source: Mol Biol Evol. 2019 Mar 7;36(6):1316–32. doi: 10.1093/molbev/msz048 (PMC6526913; doi:10.1093/molbev/msz048)
Supplement: Supplementary_Material_msz048 [file supplementary_material_msz048.zip › supplementary.pdf]

Supplementary information

Large-Scale Comparative Analysis of Codon Models Accounting for Protein and Nucleotide Selection

Iakov I. Davydov[1,2,3,*], Nicolas Salamin[1,3,†], Marc Robinson-Rechavi[2,3,†]

1. Department of Computational Biology, Biophore, University of Lausanne, 1015 Lausanne, Switzerland
2. Department of Ecology and Evolution, Biophore, University of Lausanne, 1015 Lausanne, Switzerland
3. Swiss Institute of Bioinformatics, 1015 Lausanne, Switzerland

[*]Corresponding author, iakov.davydov@gmail.com
[†]These authors contributed equally to this work.

Contents

1 Comparison of M8 and branch-site models **3**
1.1 M8 . . . 3
1.2 Branch-site model . . . 5

2 Branch-site model and background positive selection **7**

List of Tables

S1 Accuracy for all M8-based simulations . . . 9
S2 Area under curve for branch-site model simulations . . . 10
S3 Accuracy for all branch-site model simulations . . . 11
S4 Positive selection predictions (vertebrates) . . . 12
S5 Linear model of relative model support (vertebrates) . . . 13
S6 Linear model of α (vertebrates) . . . 14
S7 Positive selection prediction for a subset of the Drosophila dataset . . . 15
S8 GO-enrichment for Drosophila (with rate variation) . . . 16
S9 GO-enrichment for Drosophila (without rate variation) . . . 17
S10 List of detected genes associated with dynein in Drosophila (codon gamma rate variation) . . 18
S11 Linear model of α (Drosophila) . . . 19
S12 Linear model of relative model support (Drosophila, excluding highly recombining genes) . . 20
S13 Linear model of α (Drosophila, excluding highly recombining genes) . . . 21
S14 Linear model of relative model support (Drosophila, including proportion of branches under positive selection) . . . 22
S15 Linear model of proportion of branches identified only in the absence of rate variation (vertebrates) . . . 23
S16 Linear model of proportion of branches identified only in the absence of rate variation (Drosophila) 24
S17 Linear model of proportion of branches identified only in the absence of rate variation (Drosophila, excluding highly recombining genes) . . . 25
S18 Expression anatomical enrichment for Drosophila . . . 26
S19 Simulation parameters . . . 27
S20 Variable transformations . . . 28

List of Figures

S1 Precision versus recall (M8) . . . 29
S2 False positive rate versus significance level (M8) . . . 30
S3 True positive rate versus significance level (M8) . . . 31
S4 False positive rate versus α (M8) . . . 32
S5 ROC on the M8 dataset, true branch lengths . . . 33
S6 FPR on the M8 dataset, true branch lengths . . . 34
S7 False positive rate versus significance level (branch-site model) . . . 35
S8 Precision versus recall (branch-site model) . . . 36
S9 False positive rate versus α (branch-site model) . . . 37
S10 ROC on the branch-site dataset, true branch lengths . . . 38
S11 FPR on the branch-site dataset, true branch lengths . . . 39
S12 Phylogenetic tree (vertebrates) . . . 40
S13 Phylogenetic tree (Drosophila) . . . 41
S14 Relative rates versus distance from start codon (Drosophila) . . . 42
S15 ω in the exon-intron and intron-exon junctions (Drosophila) . . . 43
S16 ω versus distance from the start codon (Drosophila) . . . 44
S17 ρ versus distance from the start codon (Drosophila) . . . 45
S18 Distribution of simulation parameters . . . 46
S19 Distribution of α . . . 47
S20 Effect of branch length estimation . . . 48
S21 Distribution of transformed variables . . . 49
S22 Linear model residuals (vertebrates) . . . 50
S23 Linear model residuals (Drosophila) . . . 51

1 Comparison of M8 and branch-site models

We used simulations to compare the performance of M8 and branch-site models. First, we were interested if rate variation in the data inflates the false positive rate. Second, how sensitive is the model M8 in the case of data simulated using the branch-site model, i.e., if positive selection affects only a single branch. Third, if we can detect positive selection in the data simulated under the M8 model by testing individual branches with the branch-site model. In other words, if selection affecting all branches can be identified by testing branches one by one.

We used the same dataset as described in the Methods section of the main text. For the branch-site model, we tested all branches one by one and integrated results using either Fisher's method or minimum p-value.

1.1 M8

The M8 model is sensitive to variation in the substitution rate (Fig. 1). This result is similar to the behavior of the model M8 under M8 simulations (Fig. S2); false positive rate inflation is especially strong in case of codon gamma rate variation. The performance of M8 when detecting short episodes of positive selection is extremely low (Fig. 2). Thus it is not recommended to use M8 if you expect positive selection to be episodic.

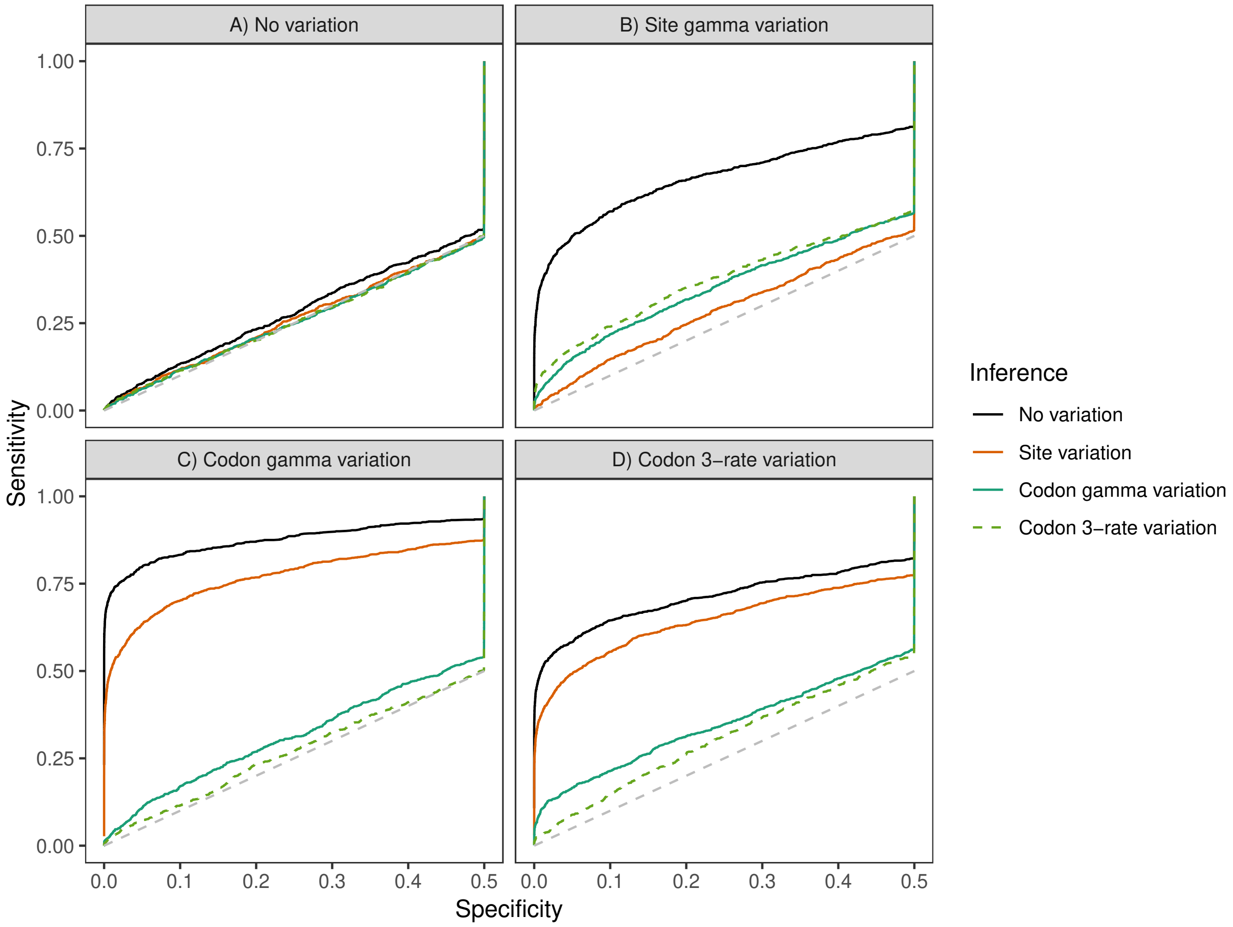

Figure 1: False positive rate as a function of significance level for four M8-based models (M8 with no rate variation, M8 with site rate variation, M8 with codon gamma rate variation, and M8 with codon 3-rate variation) on datasets simulated with the branch-site model A) without rate variation, B) with site rate variation, C) with codon gamma rate variation, and D) with codon 3-rate variation. The dashed diagonal line corresponds to the expected false-positive rate under the null hypothesis.

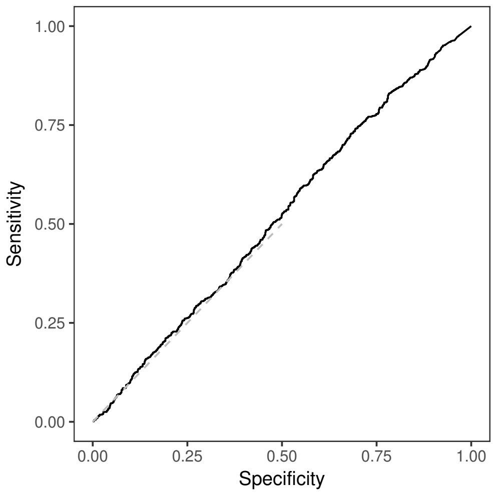

Figure 2: Receiver operating characteristic (ROC) of the model M8 (no rate variation) on simulations performed using branch-site model (no rate variation).

1.2 Branch-site model

The false positive rate of the branch-site test is not affected by rate variation (Fig. 3). Similarly, the branch-site test was less affected by rate variation in the branch-site based simulations (Fig. S7).

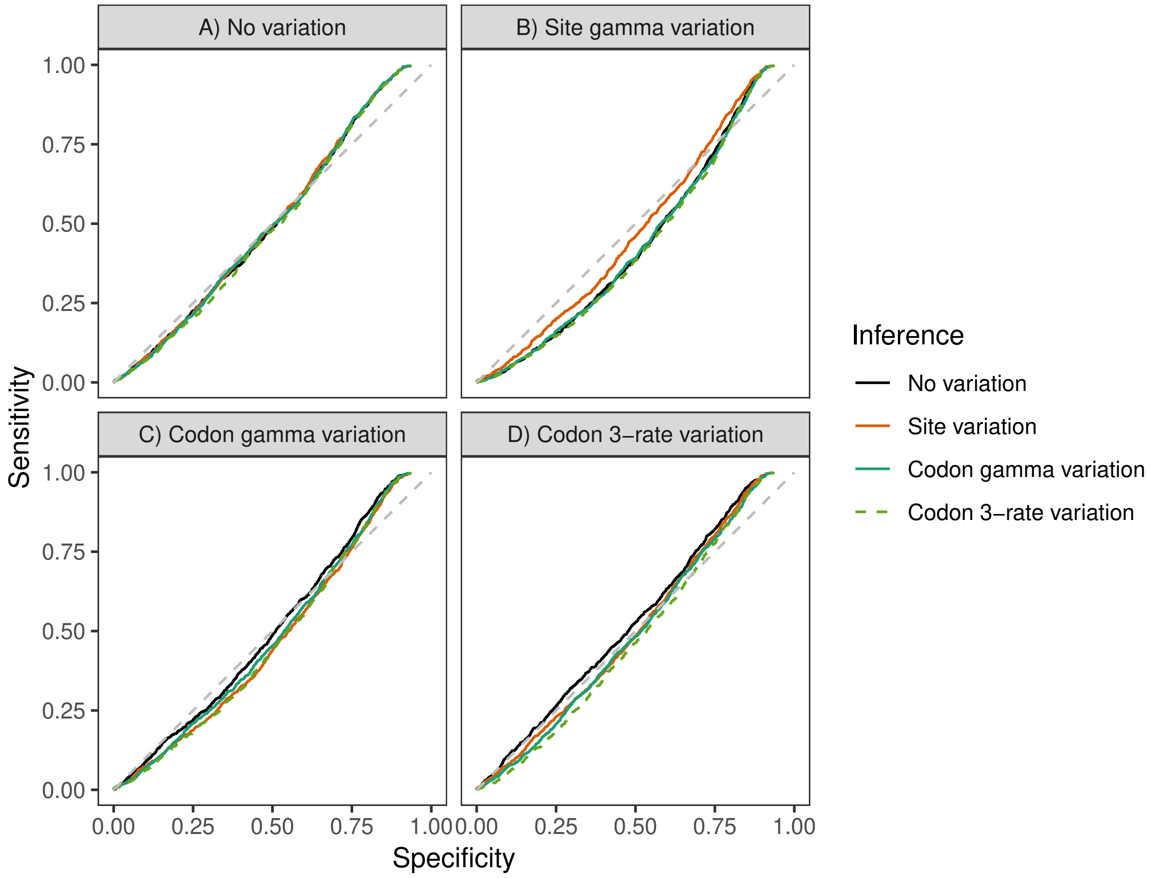

Figure 3: False positive rate as a function of significance level for four branch-site based models (branch-site model with no rate variation, branch-site model with site rate variation, branch-site model with codon gamma variation, and branch-site model with codon 3-rate rate variation) on datasets simulated under the M8 model A) without rate variation, B) with site rate variation, C) with codon gamma rate variation, and D) with codon 3-rate variation. The dashed diagonal line corresponds to the expected false-positive rate under the null hypothesis. All the branches were tested, and p-values were aggregated using Fisher's method.

The branch-site model is able to detect positive selection which is affecting all branches, as in the M8 model (Fig. 4). However, statistical performance is substantially degraded compared to the M8 model. There can be two reasons for this. First, while the branch-site model used this way tests every branch, sites evolving under positive selection do not have to be the same for different individual tests. Therefore the aggregated branch-site test, unlike M8, cannot correctly integrate a weak signal of positive selection coming from the same sites. Second, the M8 model has a more complex distribution of ω values among the sites compared to the branch-site model. This mismatch to the models' assumptions can also play a role in the statistical performance degradation.

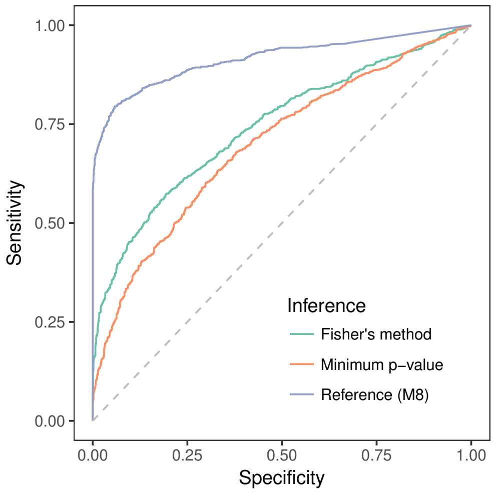

Figure 4: ROC of the branch-site model (no rate variation) on simulations performed using the model M8 (no rate variation). Two p-value aggregation methods were used: Fisher's method and minimum p-value. Inference using M8 is shown as a reference.

2 Branch-site model and background positive selection

We studied the performance of the branch-site model in the presence of strong positive selection along the background branches. For that we chose a single tree (`0176.nwk` in the git repository). We then randomly assigned some background branches to have positive selection ($\omega_3 = 20$) in 30% of alignment codon sites ($p_3 = 0.3$); sites with background positive selection were chosen at random independently for each background branch. The models used for these simulations were an extension of the branch-site model. To simulate a worst-case scenario the foreground positive selection was weaker than background positive selection ($\omega_2 = 10$), with the proportion of sites with foreground positive selection $p_2 = 0.05$. The number of branches with background positive selection was incremented from zero to fifteen (i.e., all background branches). Both the simulations and the inference were performed either with no rate variation or with codon gamma rate variation. For each number of branches with background positive selection and type of rate variation, we performed 1,000 simulations. The inference in all cases was performed on the simulated foreground branch.

We observe that the probability of foreground positive selection detection decreases with the increase in the amount of background positive selection, i.e., with the number of positively selected branches in the background (Fig. 5, 6; Pearson's $r = -0.85$, p-value$= 3.7 \cdot 10^{-5}$ and $r = -0.76$, p-value$= 0.0007$ for no variation and codon gamma rate variation, respectively). Yet, even though we simulate a large proportion of sites evolving under strong background positive selection, the decrease of the method's power is rather weak, from 56% to 47% and from 37% to 30% in the worst case for no variation and codon gamma rate variation, respectively.

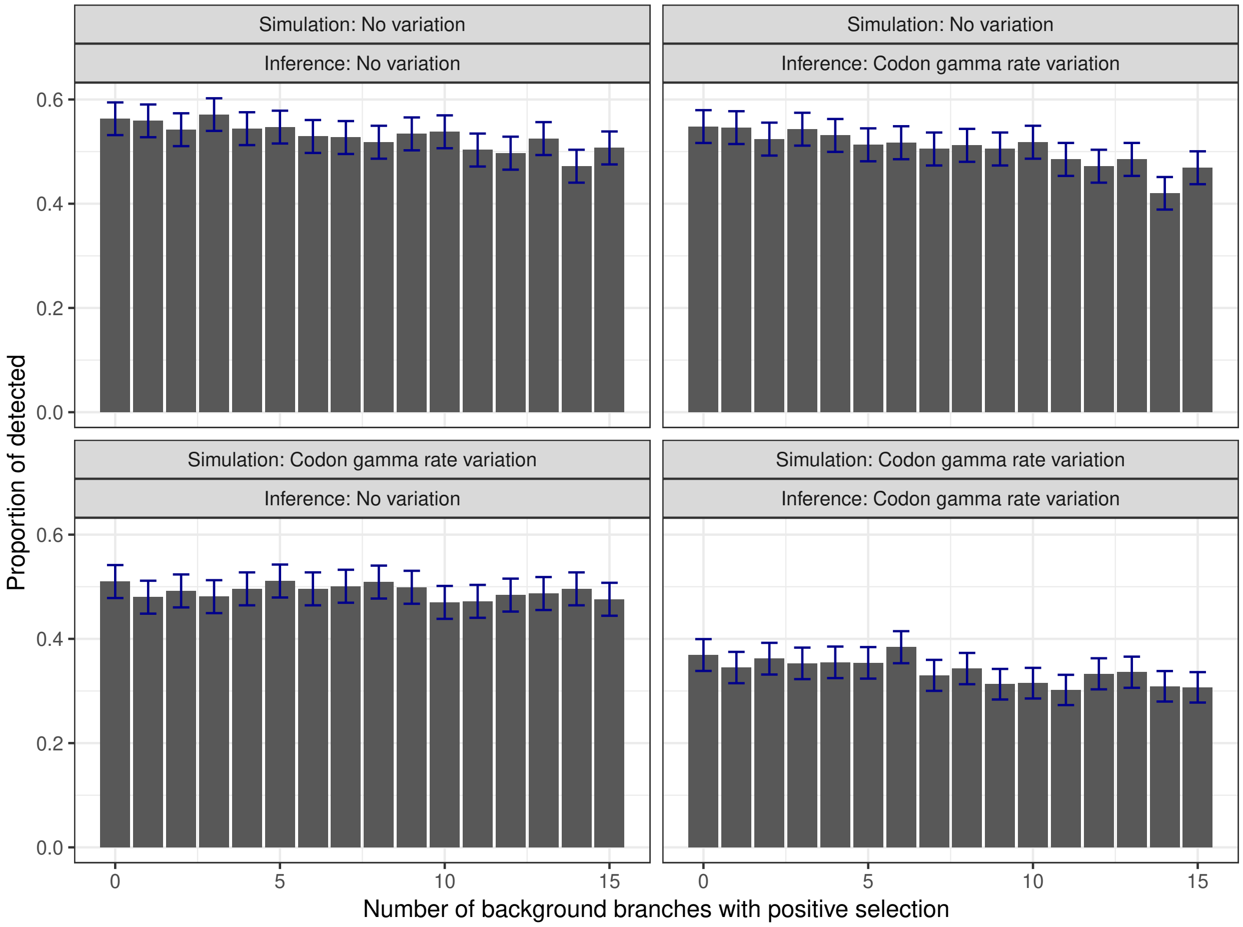

Figure 5: Proportion of simulations with foreground positive selection successfully detected as a function of the number of background branches with positive selection. Each panel corresponds to a combination of rate variation used for simulation and inference. Blue error bars show the 95% confidence intervals for population proportion (normal approximation).

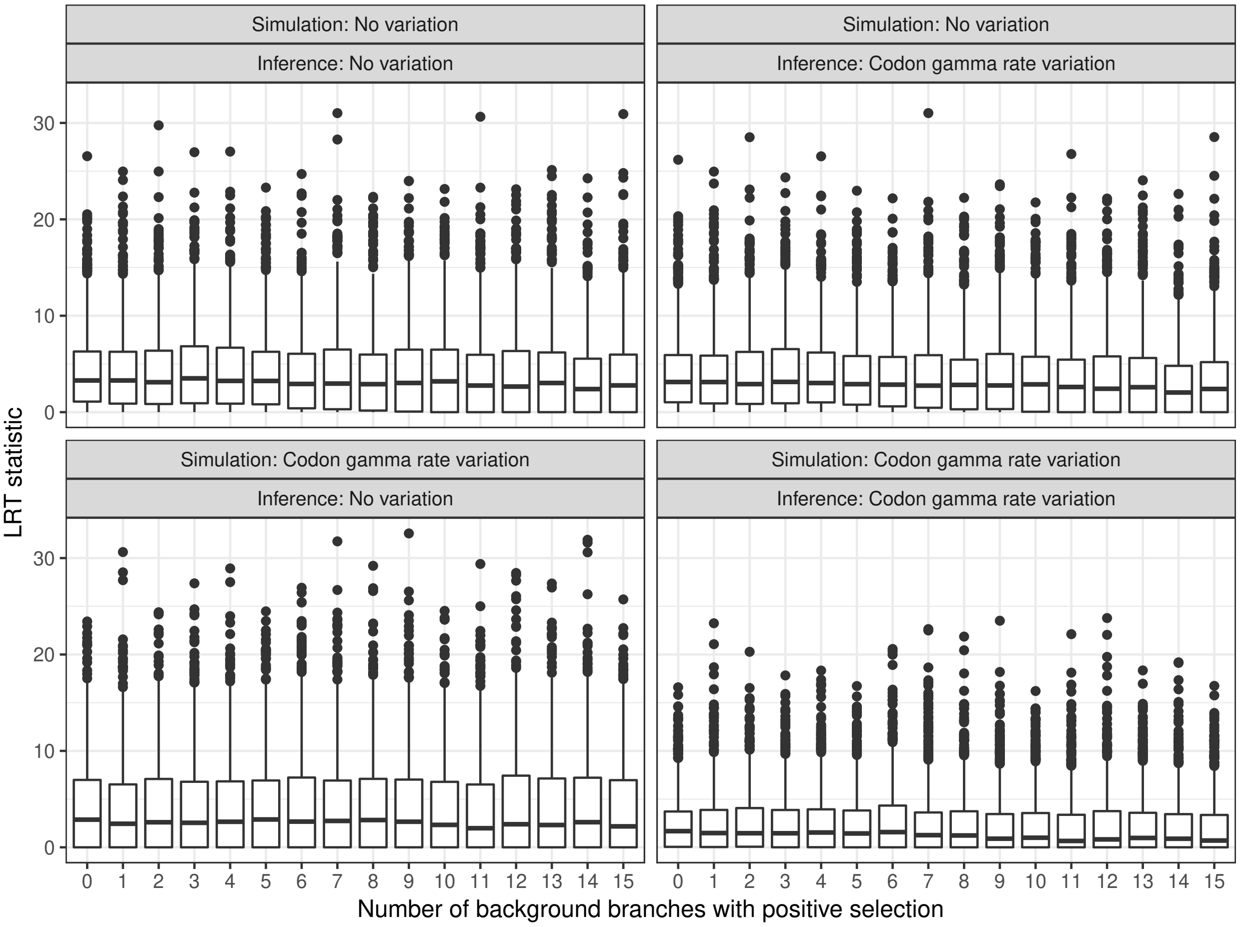

Figure 6: Value of the LRT statistic for the foreground branch as a function of the number of background branches with positive selection. Each panel corresponds to a combination of rate variation used for simulation and inference.

Tables

Estimation	Simulation: No variation	Site variation	Codon 3-rate variation	Codon gamma variation
No var.	0.856/ 100%	0.755/92.8%	0.704/88.1%	0.638/77.0%
Site var.	0.852/99.5%	0.815/ 100%	0.729/91.2%	0.694/83.8%
Codon 3-rate var.	0.806/94.2%	0.775/95.2%	0.787/98.6%	0.790/95.4%
Codon gamma var.	0.825/96.3%	0.800/98.3%	0.798/ 100%	0.829/ 100%
BUSTED	0.719/84.0%	0.669/82.2%	0.670/80.9%	0.697/87.3%

Table S1: Accuracy for all M8-based simulations and for BUSTED. Second number computed as proportion of maximum accuracy for a particular simulation (i.e., maximum value for a column).

Estimation	Simulation: No variation	Site variation	Codon 3-rate variation	Codon gamma variation
No var.	0.882/99.8%	0.842/97.2%	0.863/98.0%	0.821/94.2%
Site var.	0.880/99.6%	0.866/ 100%	0.871/98.9%	0.833/95.6%
Codon 3-rate var.	0.884/ 100%	0.841/97.1%	0.880/ 100%	0.864/99.2%
Codon gamma var.	0.875/99.0%	0.847/97.7%	0.880/ 100%	0.871/ 100%

Table S2: Area under curve (AUC) for all branch-site model-based simulations. Second number computed as proportion of maximum AUC for a particular simulation.

Estimation	No variation	Site variation	Codon 3-rate variation	Codon gamma variation
	Simulation			
No var.	0.815/99.9%	0.737/97.4%	0.795/98.2%	0.763/95.3%
Site var.	0.815/99.9%	0.756/ 100%	0.802/99.1%	0.777/97.1%
Codon 3-rate var.	0.811/ 99.4%	0.730/96.4%	0.804/99.3%	0.792/98.9%
Codon gamma var.	0.816/ 100%	0.737/97.4%	0.809/ 100%	0.801/ 100%

Table S3: Accuracy for all branch-site model-based simulations. Second number computed as proportion of maximum accuracy for a particular simulation (i.e., maximum value for a column).

**A** No variation	Best supported −	Best supported +
−	7,062	59
+	817	969

**B** Site variation	Best supported −	Best supported +
−	7,515	70
+	364	958

**C** Codon 3-rate variation	Best supported −	Best supported +
−	7,755	81
+	124	947

**D** Codon gamma variation	Best supported −	Best supported +
−	7,795	91
+	84	937

Table S4: Positive selection predictions compared to the best supported model for the vertebrate dataset. A) Branch-site without rate variation B) branch-site with site rate variation C) branch-site with codon 3-rate variation D) branch-site with codon gamma rate variation.

Variable	Estimate	Std. Error	t value	p-value
Number of sequences	0.051	0.033	1.554	0.12061
**Total branch length**	0.123	0.028	4.339	$1.66 \cdot 10^{-5}$
**Alignment length**	0.791	0.041	19.382	$< 2 \cdot 10^{-16}$
Length of coding sequence	-0.029	0.045	-0.653	0.51371
**GC content (mean)**	0.175	0.028	6.227	$8.53 \cdot 10^{-10}$
**GC content (stdev)**	-0.075	0.025	-3.014	0.00268
Total intron length	-0.016	0.030	-0.540	0.58940
Number of exons	0.052	0.048	1.080	0.28057
**Maximum expression**	0.075	0.031	2.425	0.01558
Mean expression	-0.048	0.029	-1.657	0.09803

Table S5: Linear model of relative support of model with the codon gamma rate variation; vertebrate dataset. Significant variables (p-value < 0.05) in bold. Model p-value is $< 2.2 \cdot 10^{-16}$, adjusted R^2 is 0.6247. Model formula: `Relative model support ~ Number of sequences + Total branch length + Alignment length + Length of coding sequence + GC content (mean) + GC content (stdev) + Total intron length + Number of exons + Maximum expression + Mean expression`.

Variable	Estimate	Std. Error	t value	p-value
Number of sequences	0.101	0.064	1.578	0.11513
Total branch length	0.013	0.044	0.299	0.76512
Alignment length	0.044	0.050	0.874	0.38250
Length of coding sequence	0.025	0.061	0.415	0.67848
**GC content (mean)**	-0.228	0.042	-5.402	$9.23 \cdot 10^{-8}$
**GC content (stdev)**	0.197	0.038	5.176	$3.01 \cdot 10^{-7}$
Total intron length	0.004	0.035	0.110	0.91210
Number of exons	0.019	0.064	0.293	0.76954
**Maximum expression**	-0.115	0.043	-2.672	0.00773
Mean expression	0.037	0.043	0.850	0.39578

Table S6: Linear model of α parameter of gamma distribution with the codon rate variation; vertebrate dataset. Significant variables (p-value < 0.05) in bold. Model p-value is $< 2.2 \cdot 10^{-16}$, adjusted R^2 is 0.1275. Model formula: `α ~ Number of sequences + Total branch length + Alignment length + Length of coding sequence + GC content (mean) + GC content (stdev) + Total intron length + Number of exons + Maximum expression + Mean expression`.

significance threshold of 0.05			FDR threshold of 0.1		
**A**	Codon gamma variation		**B**	Codon gamma variation	
No variation	−	+	No variation	−	+
−	6,452	36	−	7,138	2
+	607	580	+	494	41
**C**	Codon 3-rate variation		**D**	Codon 3-rate variation	
No variation	−	+	No variation	−	+
−	6,449	39	−	7,140	0
+	558	629	+	505	30
**E**	Site variation		**F**	Site variation	
No variation	−	+	No variation	−	+
−	6,457	31	−	7,140	0
+	408	779	+	397	138
**G**	Codon gamma variation		**H**	Codon gamma variation	
Site variation	−	+	Site variation	−	+
−	6,822	43	−	7,533	4
+	237	573	+	99	39

Table S7: Positive selection predictions for a random subset (1,000 genes) of the Drosophila dataset. Numbers in each cell indicate how many branches were detected (+) or not detected (-) to be evolving under positive selection by different variants of the branch-site model. A,B no rate variation versus codon gamma rate variation; C,D) no rate variation versus codon 3-rate variation; E,F) no rate variation versus site rate variation; G,H) site rate variation versus codon gamma rate variation; A,C,E,G) significance threshold of 0.05; B,D,F,H) false discovery rate threshold of 0.1.

A	GO.ID	Term	Annotated	Significant	Expected	p-value	q-value
1	GO:0036156	inner dynein arm	7	3	0.17	0.00049	0.39245
2	GO:0005587	collagen type IV trimer	2	2	0.05	0.00062	0.39245
3	GO:0005700	polytene chromosome	148	9	3.69	0.00356	1.00000
4	GO:0016442	RISC complex	5	2	0.12	0.00587	1.00000
5	GO:0016456	X chromosome located dosage compensation...	5	2	0.12	0.00587	1.00000

B	GO.ID	Term	Annotated	Significant	Expected	p-value	q-value
1	GO:0045466	R7 cell differentiation	54	7	1.37	$4.4 \cdot 10^{-5}$	0.29912
2	GO:0007605	sensory perception of sound	57	8	1.45	$8.3 \cdot 10^{-5}$	0.29912
3	GO:1900182	positive regulation of protein localization to nucleus	7	3	0.18	0.00052	0.96243
4	GO:0007403	glial cell fate determination	2	2	0.05	0.00064	0.96243
5	GO:0036159	inner dynein arm assembly	8	3	0.20	0.00082	0.96243

C	GO.ID	Term	Annotated	Significant	Expected	p-value	q-value
1	GO:0045503	dynein light chain binding	13	5	0.32	$8.7 \cdot 10^{-6}$	0.015925
2	GO:0045505	dynein intermediate chain binding	14	5	0.34	$1.3 \cdot 10^{-5}$	0.015925
3	GO:0005524	ATP binding	462	26	11.21	$3.8 \cdot 10^{-5}$	0.030900
4	GO:0051959	dynein light intermediate chain binding	12	4	0.29	0.00014	0.085418
5	GO:0008569	ATP-dependent microtubule motor activity, minus-end-directed	14	4	0.34	0.00028	0.133041

Table S8: Top GO categories for genes with positive selection detected using the branch-site model with codon rate variation (Drosophila dataset). A) Cellular component; B) Biological process; C) Molecular function.

A	GO.ID	Term	Annotated	Significant	Expected	p-value	q-value
1	GO:0005700	polytene chromosome	148	85	55.04	$8.2 \cdot 10^{-6}$	0.010414
2	GO:0035097	histone methyltransferase complex	31	18	11.53	0.00036	0.229780
3	GO:0034388	Pwp2p-containing subcomplex of 90S preribosome	6	6	2.23	0.00263	1.000000
4	GO:0000784	nuclear chromosome, telomeric region	9	8	3.35	0.00527	1.000000
5	GO:0008305	integrin complex	5	5	1.86	0.00709	1.000000

B	GO.ID	Term	Annotated	Significant	Expected	p-value	q-value
1	GO:0055088	lipid homeostasis	46	31	17.16	0.00018	1
2	GO:0043484	regulation of RNA splicing	70	25	26.12	0.00102	1
3	GO:0055114	oxidation-reduction process	293	130	109.32	0.00131	1
4	GO:0000723	telomere maintenance	24	19	8.95	0.00147	1
5	GO:0034976	response to endoplasmic reticulum stress	69	35	25.75	0.00187	1

C	GO.ID	Term	Annotated	Significant	Expected	p-value	q-value
1	GO:0005524	ATP binding	462	218	171.74	$3.0 \cdot 10^{-6}$	0.0072793
2	GO:0003682	chromatin binding	148	79	55.01	$4.2 \cdot 10^{-5}$	0.0504813
3	GO:0004003	ATP-dependent DNA helicase activity	21	15	7.81	0.00089	0.7130061
4	GO:0000166	nucleotide binding	666	295	247.57	0.00401	1.0000000
5	GO:0045503	dynein light chain binding	13	10	4.83	0.00417	1.0000000

Table S9: Top GO categories for genes with positive selection detected using the branch-site model without rate variation (Drosophila dataset). A) Cellular component; B) Biological process; C) Molecular function.

	Selectome ID	Gene ID	Gene symbol	Gene name	ω_2	positions
1	EMGT00050000003039.1	FBgn0035799	CG14838	uncharacterized protein	6.62	247, 726, 738, 741, 938, 1000
2	EMGT00180000072377.2	FBgn0013813	Dhc98D	Dynein heavy chain at 89D	5.51	964, 1184, 1422, 1553, 1828, 2108, 2226, 2311, 2541, 3329, 3646, 4031, 4039, 4565, 4687, 4933
3	EMGT00180000072377.2	FBgn0013813	Dhc98D	Dynein heavy chain at 89D	4.29	388, 1166, 1181, 1986, 4214, 4762, 5071
4	EMGT00180000072377.4	FBgn0039510	CG3339	CG3339	8.80	716, 1029, 1581, 1707
5	EMGT00180000072378.1	FBgn0013811	Dhc62B	Dynein heavy chain at 62B	3.08	35, 1967, 2481
6	EMGT00180000072378.8	FBgn0013810	Dhc36C	Dynein heavy chain at 36C	4.46	27, 227, 242, 443, 488, 521, 822, 848, 1337, 1527, 1980, 2939, 2946, 3668, 4118

Table S10: A list of genes under positive selection detected by the branch-site model with codon gamma rate variation (Drosophila dataset). Amino acid positions with posterior probability > 0.9 are specified as in Selectome release 6 alignments.

Variable	Estimate	Std. Error	t value	p-value
**Number of sequences**	-0.291	0.022	-13.193	$< 2 \cdot 10^{-16}$
**Total branch length**	0.103	0.022	4.644	$3.48 \cdot 10^{-6}$
Alignment length	-0.125	0.071	-1.772	0.0765
Length of coding sequence	0.092	0.068	1.355	0.1755
**GC content (mean)**	-0.106	0.017	-6.247	$4.39 \cdot 10^{-10}$
GC content (stdev)	-0.023	0.013	-1.760	0.0784
**Total intron length**	-0.133	0.019	-6.995	$2.87 \cdot 10^{-12}$
Number of exons	-0.000	0.021	-0.015	0.9883
Maximum expression	0.008	0.030	0.265	0.7910
Mean expression	-0.024	0.029	-0.823	0.4103
**Recombination rate**	-0.052	0.010	-5.054	$4.43 \cdot 10^{-7}$

Table S11: Linear model of α parameter of gamma distribution with the codon rate variation; Drosophila dataset. Significant variables (p-value < 0.05) in bold. Model p-value is $< 2.2 \cdot 10^{-16}$, adjusted R^2 is 0.1498. Model formula: `α ~ Number of sequences + Total branch length + Alignment length + Length of coding sequence + GC content (mean) + GC content (stdev) + Total intron length + Number of exons + Maximum expression + Mean expression + Recombination rate`.

Variable	Estimate	Std. Error	t value	p-value
**Number of sequences**	0.177	0.010	17.430	$< 2 \cdot 10^{-16}$
**Total branch length**	0.118	0.012	9.683	$< 2 \cdot 10^{-16}$
**Alignment length**	0.600	0.034	17.446	$< 2 \cdot 10^{-16}$
**Length of coding sequence**	0.104	0.034	3.099	0.001952
**GC content (mean)**	0.089	0.010	8.651	$< 2 \cdot 10^{-16}$
GC content (stdev)	0.008	0.009	0.883	0.377265
**Total intron length**	-0.046	0.014	-3.364	0.000773
**Number of exons**	0.152	0.019	8.144	$4.67 \cdot 10^{-16}$
Maximum expression	0.036	0.021	1.683	0.092409
Mean expression	-0.015	0.021	-0.706	0.480022
**Recombination rate**	0.070	0.011	6.327	$2.70 \cdot 10^{-10}$

Table S12: Linear model of relative support of model with the codon rate variation; Drosophila dataset with 30% of the observations with the highest recombination rates excluded. Significant variables (p-value < 0.05) in bold. Model p-value is $< 2.2 \cdot 10^{-16}$, adjusted R^2 is 0.6407. Model formula: `Relative model support ~ Number of sequences + Total branch length + Alignment length + Length of coding sequence + GC content (mean) + GC content (stdev) + Total intron length + Number of exons + Maximum expression + Mean expression + Recombination rate`.

Variable	Estimate	Std. Error	t value	p-value
**Number of sequences**	-0.273	0.025	-10.714	$< 2 \cdot 10^{-16}$
**Total branch length**	0.096	0.024	3.951	$7.88 \cdot 10^{-5}$
Alignment length	-0.140	0.087	-1.616	0.106
Length of coding sequence	0.095	0.083	1.144	0.253
**GC content (mean)**	-0.113	0.020	-5.668	$1.52 \cdot 10^{-8}$
GC content (stdev)	-0.021	0.015	-1.371	0.171
**Total intron length**	-0.125	0.023	-5.552	$2.95 \cdot 10^{-8}$
Number of exons	0.006	0.024	0.231	0.817
Maximum expression	-0.014	0.035	-0.400	0.689
Mean expression	-0.006	0.036	-0.181	0.857
**Recombination rate**	-0.064	0.016	-3.976	$7.08 \cdot 10^{-5}$

Table S13: Linear model of α parameter of gamma distribution with the codon rate variation; Drosophila dataset with 30% of the observations with the highest recombination rates excluded. Significant variables (p-value < 0.05) in bold. Model p-value is $< 2.2 \cdot 10^{-16}$, adjusted R^2 is 0.1411. Model formula: `α ~ Number of sequences + Total branch length + Alignment length + Length of coding sequence + GC content (mean) + GC content (stdev) + Total intron length + Number of exons + Maximum expression + Mean expression + Recombination rate`.

Variable	Estimate	Std. Error	t value	p-value
**Number of sequences**	0.194	0.008	22.865	$< 2 \cdot 10^{-16}$
**Total branch length**	0.094	0.011	8.664	$< 2 \cdot 10^{-16}$
**Alignment length**	0.569	0.030	19.010	$< 2 \cdot 10^{-16}$
**Length of coding sequence**	0.128	0.029	4.366	$1.28 \cdot 10^{-5}$
**GC content (mean)**	0.081	0.009	9.456	$< 2 \cdot 10^{-16}$
GC content (stdev)	0.010	0.007	1.380	0.1676
**Total intron length**	-0.052	0.011	-4.566	$5.03 \cdot 10^{-6}$
**Number of exons**	0.157	0.015	10.250	$< 2 \cdot 10^{-16}$
**Maximum expression**	0.036	0.018	2.039	0.0414
Mean expression	-0.021	0.018	-1.161	0.2455
**Recombination rate**	0.057	0.007	8.267	$< 2 \cdot 10^{-16}$
**Proportion of branches with positive selection**	0.056	0.009	6.477	$9.93 \cdot 10^{-11}$

Table S14: Linear model of relative support of model with codon rate variation; Drosophila dataset. Explanatory variables include the proportion of branches with positive selection detected using the branch-site model with codon gamma rate variation. Significant variables (p-value < 0.05) in bold. Model p-value is $< 2.2 \cdot 10^{-16}$, adjusted R^2 is 0.6505. Model formula: `Relative model support ~ Number of sequences + Total branch length + Alignment length + Length of coding sequence + GC content (mean) + GC content (stdev) + Total intron length + Number of exons + Maximum expression + Mean expression + Recombination rate + Proportion of branches with positive selection`.

Variable	Estimate	Std. Error	t value	p-value
**Number of sequences**	-0.122	0.034	-3.569	0.000385
**Total branch length**	0.156	0.038	4.075	$5.17 \cdot 10^{-5}$
**Alignment length**	0.154	0.045	3.376	0.000778
Length of coding sequence	0.033	0.066	0.500	0.617029
**GC content (mean)**	0.168	0.046	3.686	0.000246
GC content (stdev)	0.031	0.038	0.826	0.409009
Total intron length	-0.057	0.057	-1.000	0.317704
Number of exons	-0.038	0.071	-0.537	0.591548
Maximum expression	-0.076	0.052	-1.468	0.142588
Mean expression	0.013	0.050	0.253	0.800026

Table S15: Linear model of proportion of branches for an alignment identified to be under positive selection by the branch-site model without rate variation, but not identified using branch-site model with codon rate variation, vertebrate dataset. Significant variables (p-value < 0.05) in bold. Model p-value= $3.036 \cdot 10^{-16}$, adjusted R^2 is 0.1231. Model formula: `Proportion of false positives ~ Number of sequences + Total branch length + Alignment length + Length of coding sequence + GC content (mean) + GC content (stdev) + Total intron length + Number of exons + Maximum expression + Mean expression`.

Variable	Estimate	Std. Error	t value	p-value
Number of sequences	0.007	0.012	0.532	0.59489
**Total branch length**	0.241	0.014	17.376	$< 2 \cdot 10^{-16}$
**Alignment length**	0.207	0.037	5.656	$1.60 \cdot 10^{-8}$
**Length of coding sequence**	0.083	0.037	2.213	0.02689
**GC content (mean)**	0.044	0.011	3.828	0.00013
GC content (stdev)	-0.021	0.011	-1.899	0.05766
Total intron length	0.029	0.017	1.743	0.08140
**Number of exons**	-0.095	0.020	-4.801	$1.61 \cdot 10^{-6}$
**Maximum expression**	0.063	0.030	2.064	0.03902
Mean expression	-0.048	0.030	-1.630	0.10315
**Recombination rate**	0.048	0.011	4.494	$7.08 \cdot 10^{-6}$

Table S16: Linear model of proportion of branches for an alignment identified to be under positive selection by the branch-site model without rate variation, but not identified using branch-site model with codon rate variation, Drosophila dataset. Significant variables (p-value < 0.05) in bold. Model p-value is $< 2.2 \cdot 10^{-16}$, adjusted R^2 is 0.113. Model formula: `Proportion of false positives ~ Number of sequences + Total branch length + Alignment length + Length of coding sequence + GC content (mean) + GC content (stdev) + Total intron length + Number of exons + Maximum expression + Mean expression + Recombination rate`.

Variable	Estimate	Std. Error	t value	p-value
Number of sequences	-0.000	0.015	-0.019	0.98481
**Total branch length**	0.239	0.016	14.866	$< 2 \cdot 10^{-16}$
**Alignment length**	0.197	0.044	4.509	$6.64 \cdot 10^{-6}$
**Length of coding sequence**	0.102	0.044	2.295	0.02176
**GC content (mean)**	0.042	0.014	2.982	0.00287
GC content (stdev)	-0.011	0.013	-0.789	0.42998
Total intron length	0.021	0.019	1.078	0.28107
**Number of exons**	-0.100	0.023	-4.376	$1.23 \cdot 10^{-5}$
Maximum expression	0.046	0.036	1.281	0.20010
Mean expression	-0.036	0.035	-1.032	0.30234
**Recombination rate**	0.075	0.017	4.288	$1.83 \cdot 10^{-5}$

Table S17: Linear model of proportion of branches for an alignment identified to be under positive selection by the branch-site model without rate variation, but not identified using branch-site model with codon rate variation; Drosophila dataset with 30% of the observations with the highest recombination rates excluded. Significant variables (p-value < 0.05) in bold. Model p-value is $< 2.2 \cdot 10^{-16}$, adjusted R^2 is 0.115. Model formula: `Proportion of false positives ~ Number of sequences + Total branch length + Alignment length + Length of coding sequence + GC content (mean) + GC content (stdev) + Total intron length + Number of exons + Maximum expression + Mean expression + Recombination rate`.

A	ID	Anatomical entity	Annotated	Significant	Expected	p-value	q-value
FBbt:00003023	adult abdomen (Drosophila)	4773	1871	1747.54	1.07	$1.98 \cdot 10^{-9}$	$5.04 \cdot 10^{-7}$
FBbt:00001684	embryonic/larval hemocyte (Drosophila)	4574	1789	1674.68	1.07	$3.48 \cdot 10^{-8}$	0.00000444
UBERON:0000473	testis	5903	2258	2161.27	1.04	1e-7	0.00000852
UBERON:0001054	Malpighian tubule	4706	1829	1723.01	1.06	$2.47 \cdot 10^{-7}$	0.0000158
UBERON:0003917	arthropod fat body	5358	2060	1961.73	1.05	$4.7 \cdot 10^{-7}$	0.000024
CL:0000023	oocyte	4400	1713	1610.98	1.06	$8.89 \cdot 10^{-7}$	0.0000378
FBbt:00001778	wing disc (Drosophila)	4625	1791	1693.36	1.06	0.000002	0.000073
FBbt:00003143	adult hindgut (Drosophila)	4898	1880	1793.31	1.05	0.0000164	0.000522
FBbt:00000016	thoracic segment (Drosophila)	5070	1974	1856.29	1.06	0.000234	0.00662
UBERON:0000972	antenna	6057	2277	2217.66	1.03	0.000561	0.0143
FBbt:00003133	crop (Drosophila)	4741	1800	1735.83	1.04	0.00119	0.0254
FBbt:00001768	eye disc (Drosophila)	4730	1796	1731.8	1.04	0.0012	0.0254
FBbt:00003138	adult midgut (Drosophila)	4284	1632	1568.51	1.04	0.00157	0.0309
UBERON:0000994	spermathecum	4318	1640	1580.95	1.04	0.00301	0.0548
UBERON:0001017	central nervous system	5708	2139	2089.88	1.02	0.00556	0.0944
**B**	**ID**	**Anatomical entity**	**Annotated**	**Significant**	**Expected**	**p-value**	**q-value**
FBbt:00001684	embryonic/larval hemocyte (Drosophila)	4574	140	111.65	1.25	0.0000132	0.00337
UBERON:0000473	testis	5903	167	144.08	1.16	0.0000338	0.0043

Table S18: Top anatomical expression enrichment entities for genes with positive selection detected (Drosophila). A) Branch-site model without rate variation B) Branch-site model with codon rate variation. Only anatomical entities with q-value < 0.1 are shown.

Parameter	Distribution
Number of sequences	DiscreteUniform(8, 12)
Alignment length (in codons)	DiscreteUniform(100, 400)
κ (transition/transversion ratio)	Exponential(1)
α (gamma distribution)	0.1 + Exponential(1)
codon 3-rate variation	
p_1	Uniform(0, 1)
p_2	Uniform(0, 1)
R_1	1/(1+Exponential(1/3))
R_1	1+Exponential(1/3)
M8	
Total branch length	Exponential(1)
ω (positive selection)	Gamma(5, 1)
p	0.2 + Exponential(2)
q	0.2 + Exponential(2)
p_0	Beta(8, 2)
Branch-site	
Total branch length	10 $\times$ Exponential(1)
ω_0	Beta(1, 5)
$p_0 + p_1$	Beta(10, 1.5)
$\frac{p_0}{p_0+p_1}$	Beta(8, 2)
ω_2 (positive selection)	Gamma(50, 1)

Table S19: Statistical distributions used for simulation parameters.

Parameter	Transformation
Total intron length	$\log(x+1)$
Number of exons	$\sqrt{x}$
Maximum expression	$\log x$
Mean expression	$\log x$
Length of coding sequence	$\log x$
Total branch length	$\log x$
Alignment length (codons)	$\log x$
Relative model support	$\sqrt{\max(x,0)}$
α (gamma distribution)	$\log x$
ω_0 (branch-site model)	$\log x$
Recombination rate	$\sqrt{x}$

Table S20: Variable transformations used for the linear model.

Figures

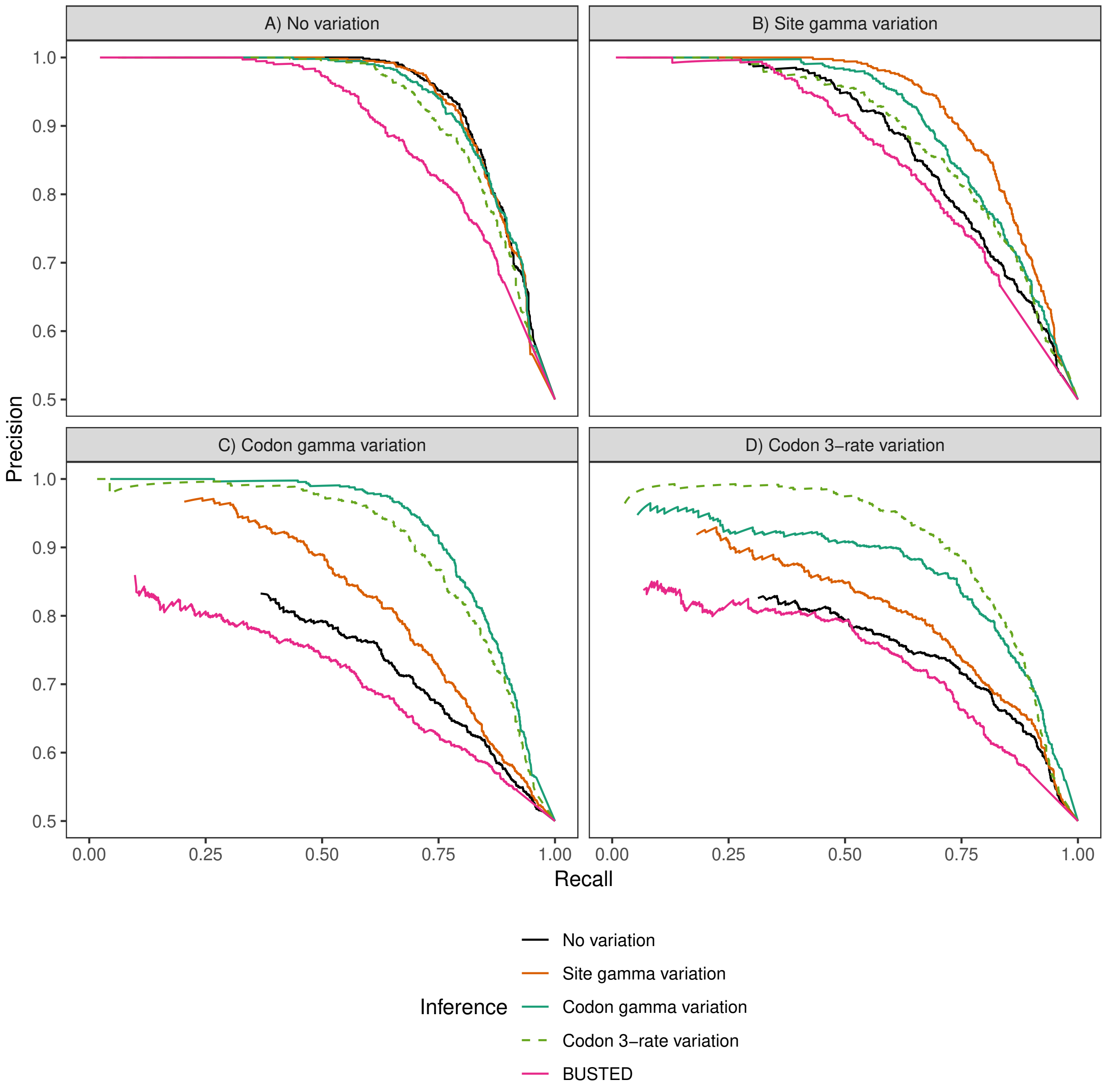

Figure S1: Precision as a function of recall for four M8-based models (M8 with no rate variation, M8 with site rate variation, M8 with codon gamma rate variation, and M8 with codon 3-rate variation) and for BUSTED on datasets A) without rate variation, B) with site rate variation, C) with codon gamma rate variation, and D) with codon 3-rate variation.

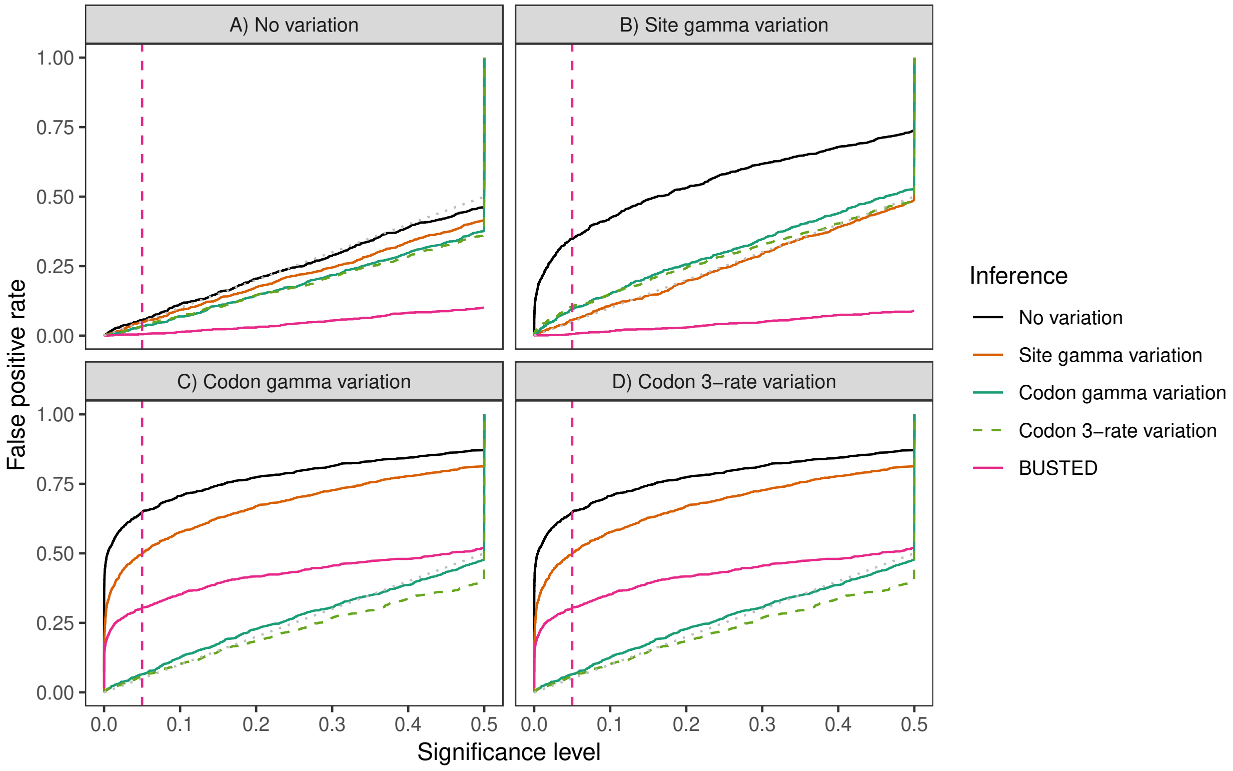

Figure S2: False positive rate as a function of significance level for four M8-based models (M8 with no rate variation, M8 with site rate variation, M8 with codon gamma rate variation, and M8 with codon 3-rate variation) and for BUSTED on datasets A) without rate variation, B) with site rate variation, C) with codon gamma rate variation, and D) with codon 3-rate variation. Vertical dashed line indicates 0.05 threshold. Dashed diagonal line corresponds to the expected false-positive rate under the null hypothesis.

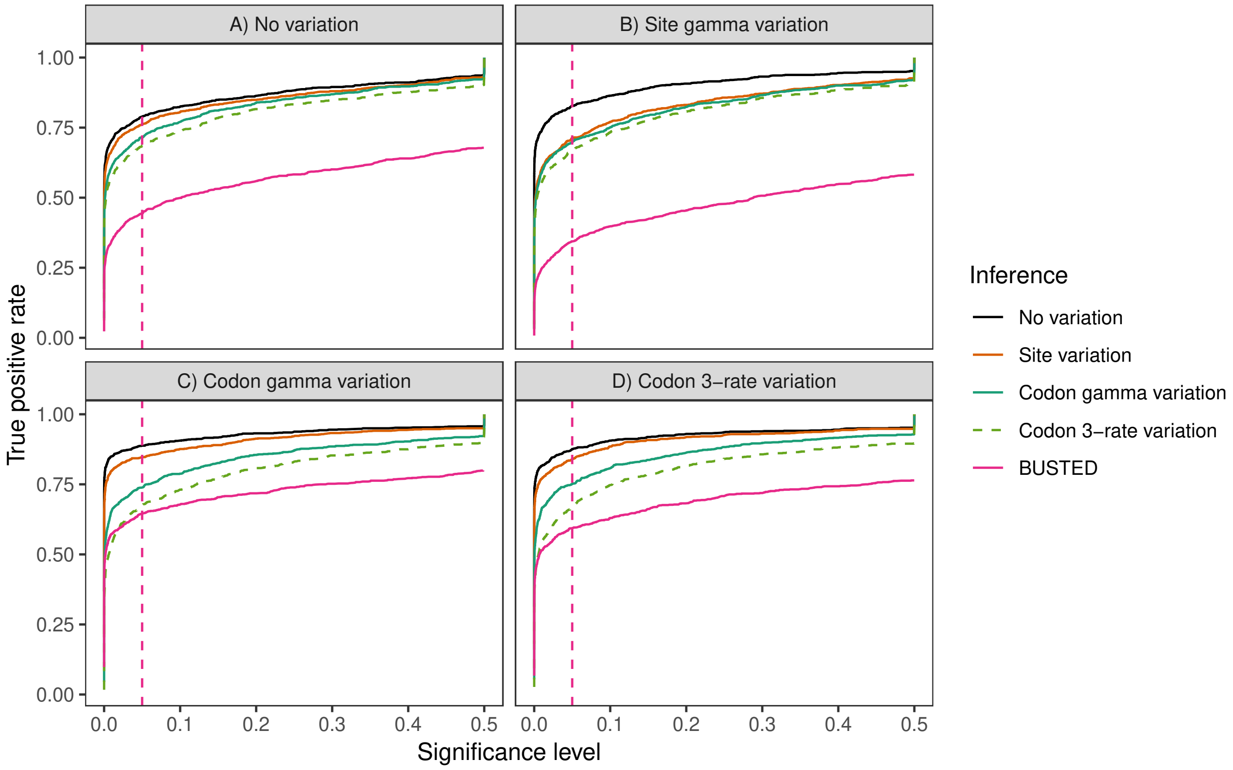

Figure S3: True positive rate (probability of detection) as a function of significance level for four M8-based models (M8 with no rate variation, M8 with site rate variation, M8 with codon gamma rate variation, and M8 with codon 3-rate variation) and for BUSTED on datasets A) without rate variation, B) with site rate variation C) with codon gamma rate variation, and D) with codon 3-rate variation. Vertical dashed line indicates 0.05 threshold.

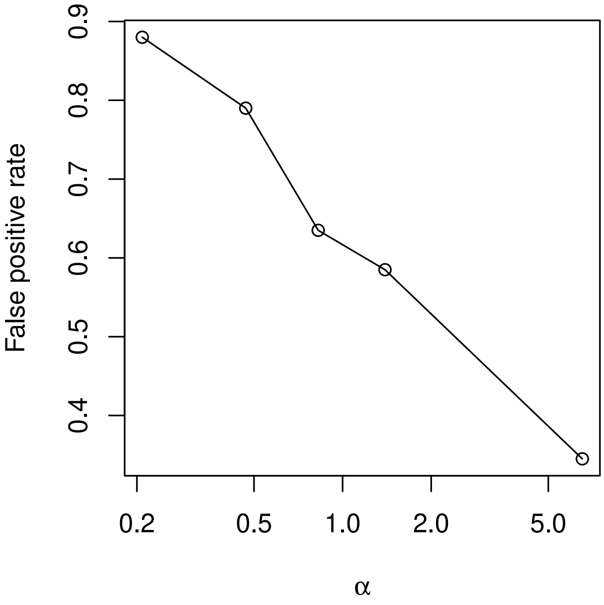

Figure S4: False positive rate as a function of α (gamma distribution parameter, log scale). Smaller α corresponds to stronger rate variation. The data was simulated using the M8 model with codon rate variation and estimated using the M8 model with no rate variation. The data was split into five equal parts according to quantiles of α.

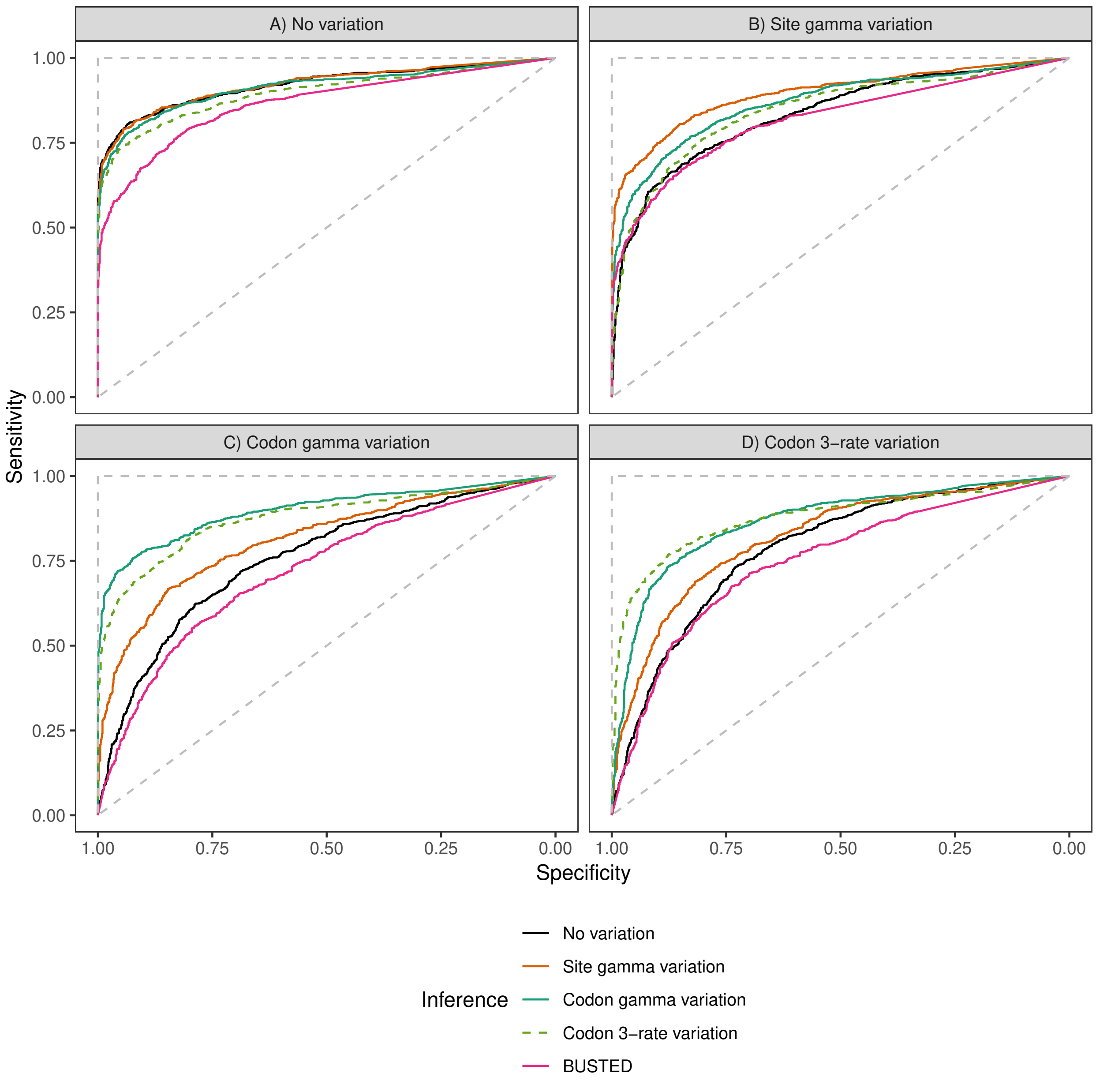

Figure S5: Performance (ROC) of four M8-based models (M8 with no rate variation, M8 with site rate variation, M8 with codon codon rate variation, and M8 with codon 3-rate variation) and of BUSTED on datasets A) without rate variation, B) with site rate variation, C) with codon gamma rate variation, and D) with codon 3-rate variation. True branch lengths were used during estimation. The pink dashed line indicates the 0.95 specificity threshold (i.e., false positive rate of 0.05). The dashed diagonal line shows theoretical performance of the random predictor, the dashed vertical and horizontal lines indicate theoretical performance of the perfect predictor.

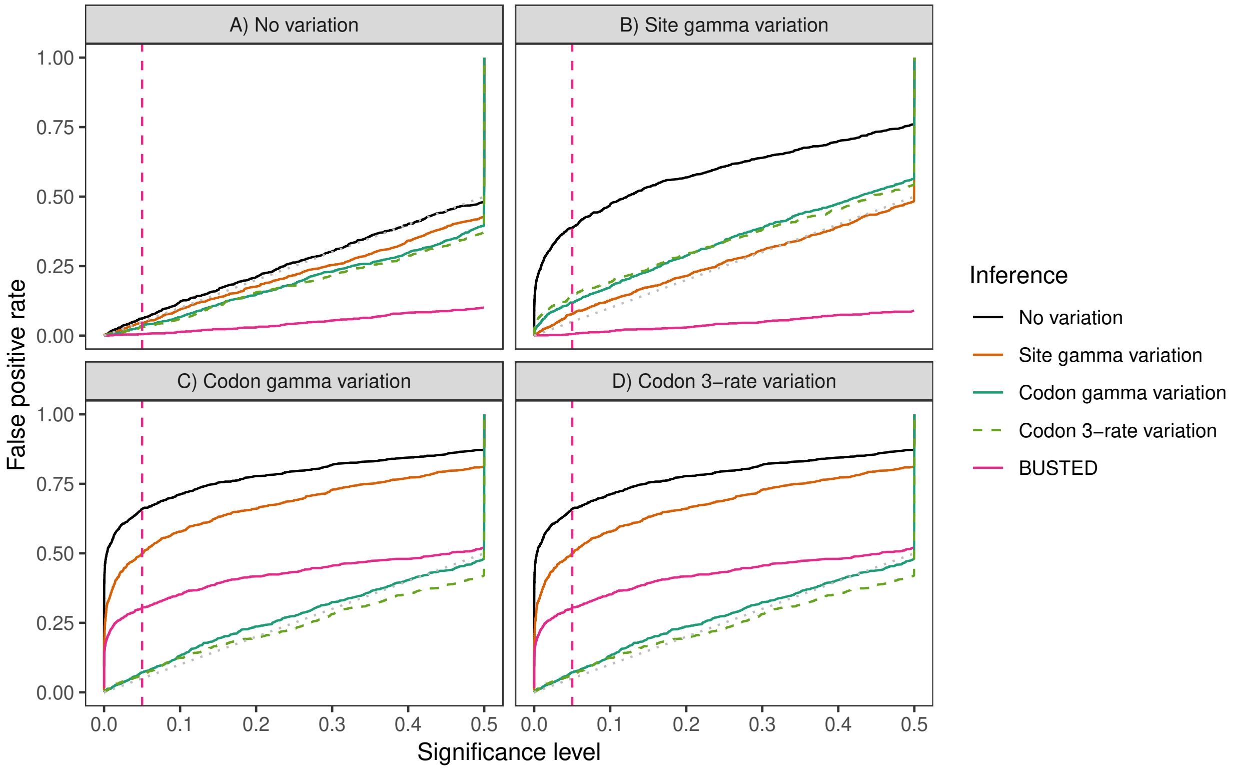

Figure S6: False positive rate as a function of significance level for four M8-based models (M8 with no rate variation, M8 with site rate variation, M8 with codon gamma rate variation, and M8 with codon 3-rate variation) and for BUSTED on datasets A) without rate variation, B) with site rate variation, C) with codon gamma rate variation, and D) with codon 3-rate variation. True branch lengths were used during estimation. Vertical dashed line indicates 0.05 threshold. Dashed diagonal line corresponds to the expected false-positive rate under the null hypothesis.

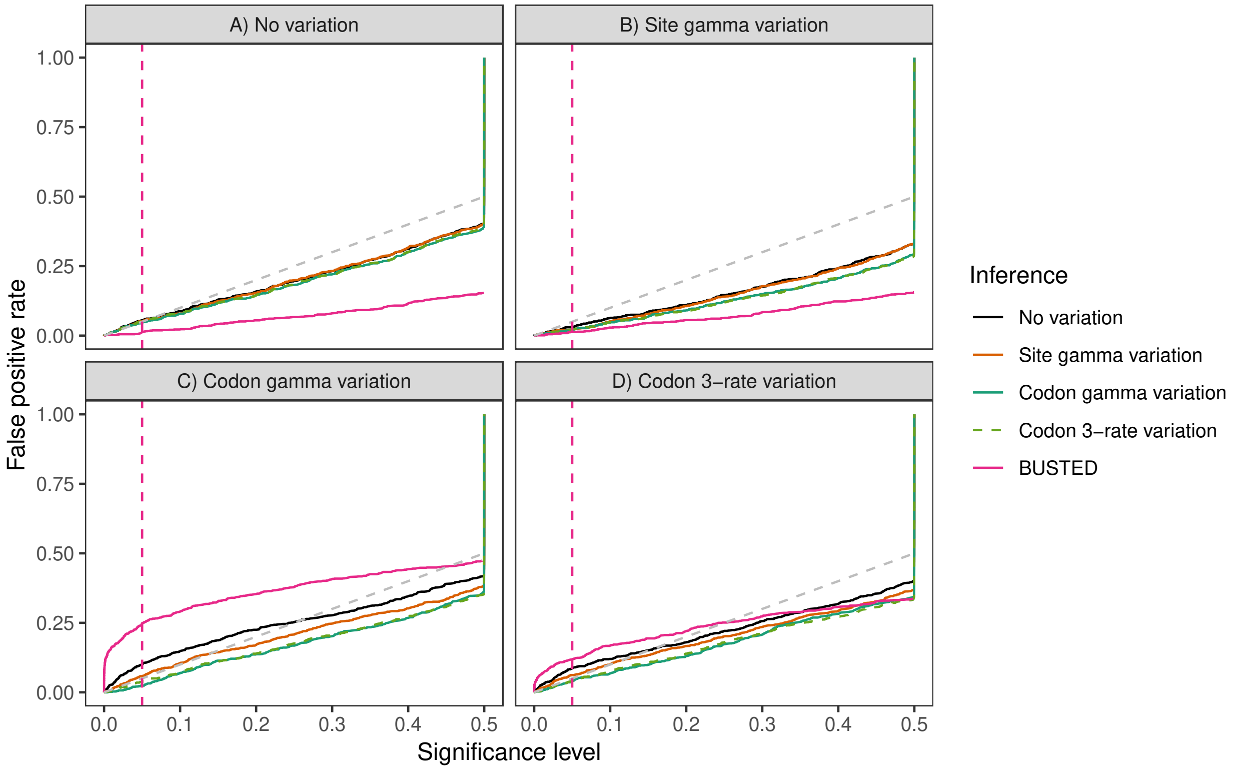

Figure S7: False positive rate as a function of significance level for four branch-site models (branch-site with no rate variation, branch-site with site rate variation, branch-site with codon gamma rate variation, and branch-site with codon 3-rate variation) and for BUSTED on datasets A) without rate variation, B) with site rate variation, C) with codon gamma rate variation, and D) with codon 3-rate variation. Vertical dashed line indicates 0.05 threshold. Dashed diagonal line corresponds to the expected false-positive rate under the null hypothesis.

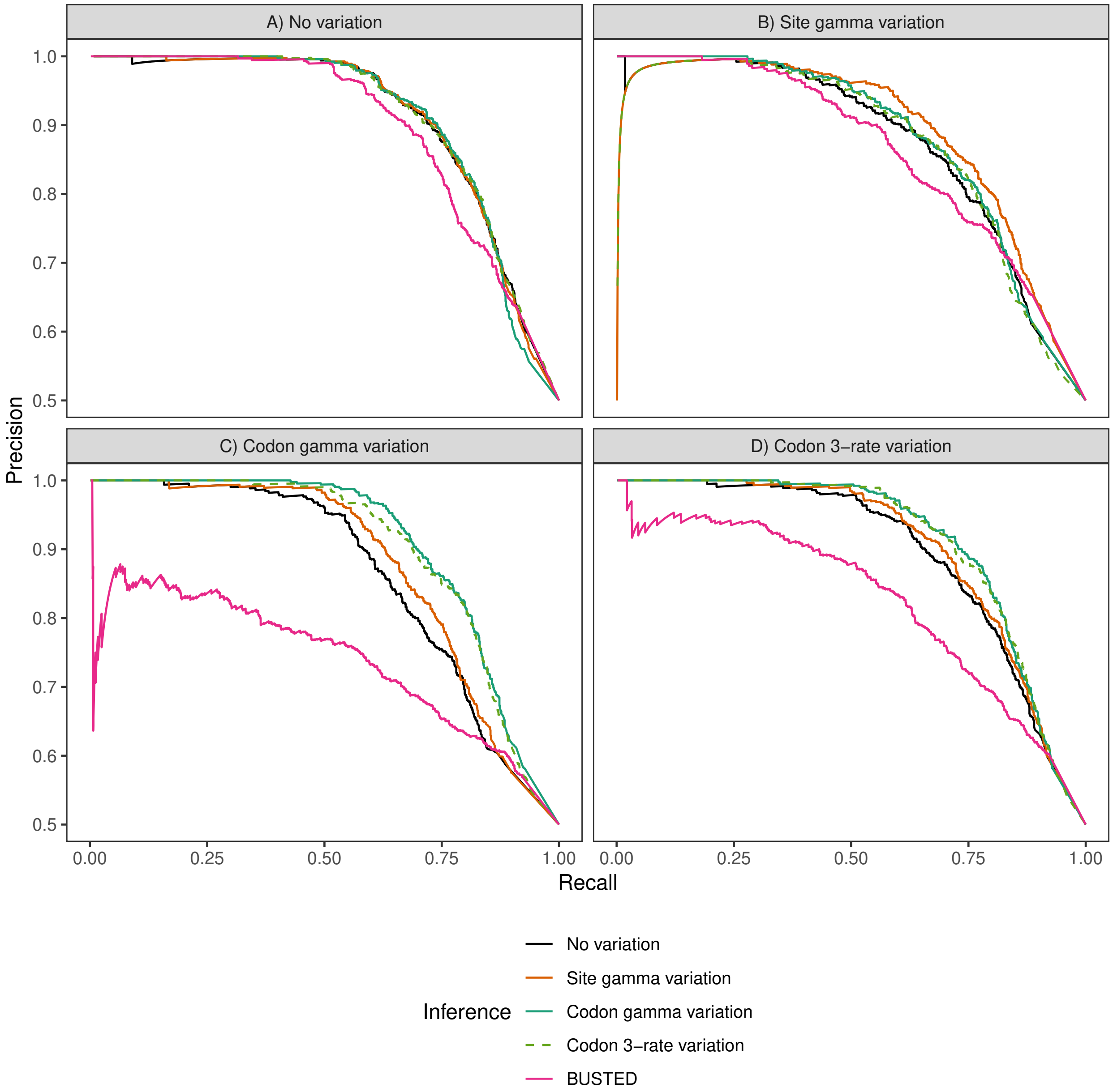

Figure S8: Precision as a function of recall for four branch-site models (branch-site with no rate variation, branch-site with site rate variation, branch-site with codon gamma rate variation, and branch-site with codon 3-rate variation) and for BUSTED on datasets A) without rate variation, B) with site rate variation, C) with codon gamma rate variation, and D) with codon 3-rate variation.

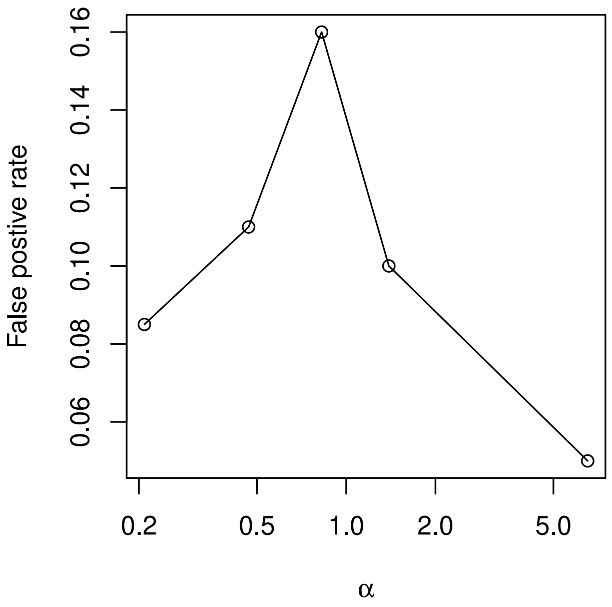

Figure S9: False positive rate as a function of α (gamma distribution parameter, log scale). Smaller α corresponds to stronger rate variation. The data was simulated using the branch-site model with codon rate variation and estimated using the branch-site with no rate variation. The data was split into five equal parts according to quantiles of α.

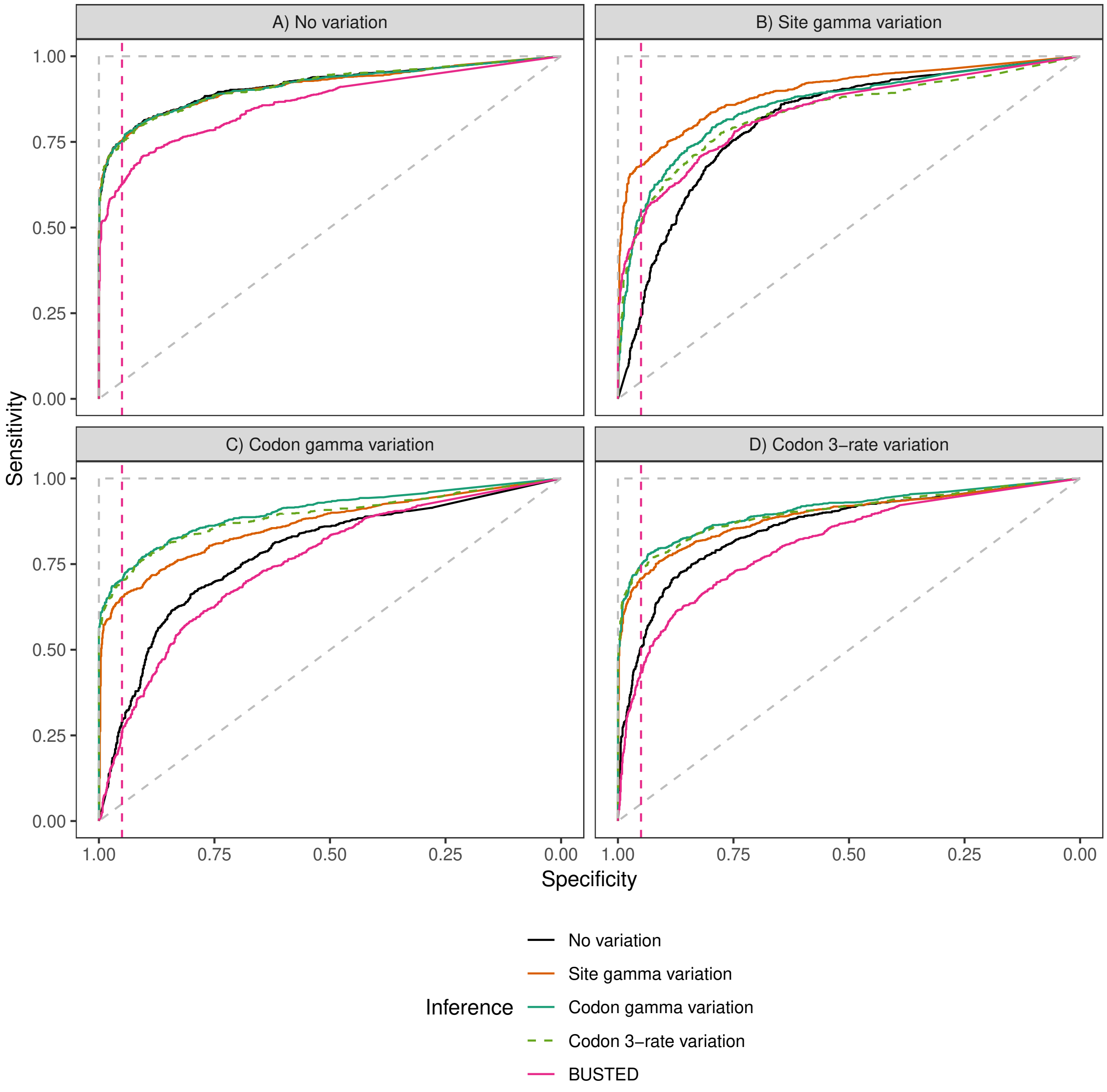

Figure S10: Performance (ROC) of four branch-site-based models (branch-site with no rate variation, branch-site with site rate variation, branch-site with codon 3-rate variation, and branch-site with codon gamma rate variation) and of BUSTED on datasets A) without rate variation, B) with site rate variation, C) with codon gamma rate variation, and D) with codon 3-rate variation. True branch lengths were used during estimation. The pink dashed line indicates the 0.95 specificity threshold (i.e., false positive rate of 0.05). The dashed diagonal line shows theoretical performance of the random predictor, the dashed vertical and horizontal lines indicate theoretical performance of the perfect predictor.

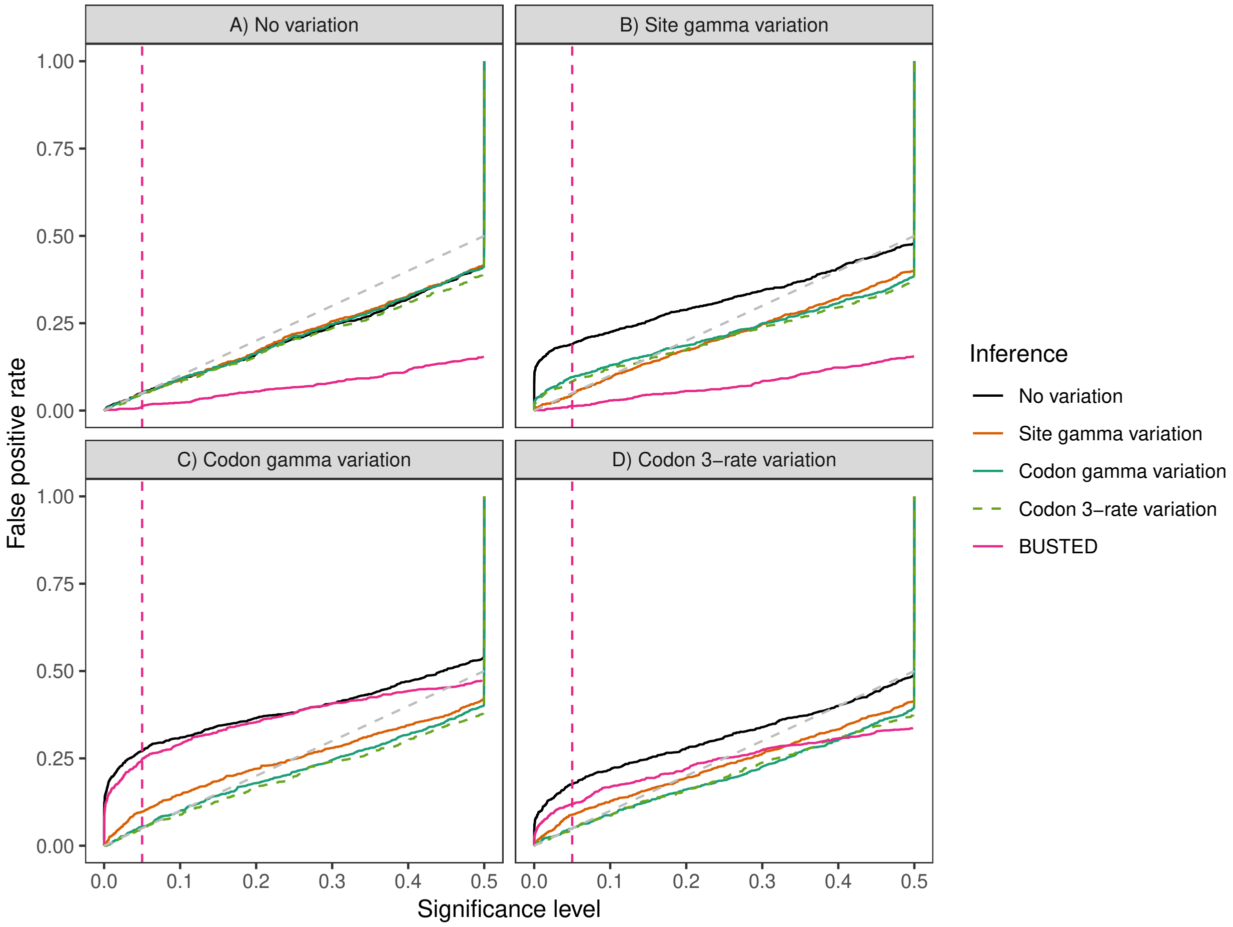

Figure S11: False positive rate as a function of significance level for four branch-site-based models (branch-site model with no rate variation, branch-site model with site rate variation, branch-site model with codon gamma rate variation, and branch-site model with codon 3-rate variation) and for BUSTED on datasets A) without rate variation, B) with site rate variation, C) with codon gamma rate variation, and D) with codon 3-rate variation. True branch lengths were used during estimation. Vertical dashed line indicates 0.05 threshold. Dashed diagonal line corresponds to the expected false-positive rate under the null hypothesis.

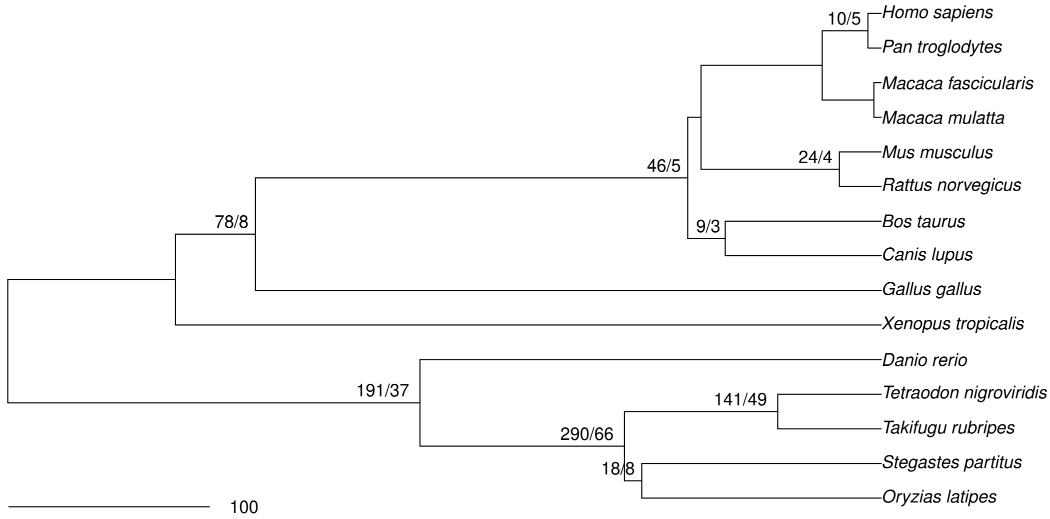

Figure S12: Phylogenetic tree of vertebrate species used in the study. Tree topology and branch lengths in millions of years were acquired from TimeTree (`http://www.timetree.org/`). Labels indicate the number of genes identified as evolving under positive selection at a particular branch (FDR threshold 0.1). The first numbers correspond to the branch-site model without rate variation, the second numbers correspond to the branch-site model with codon gamma rate variation. Only tests performed on branches compatible between the gene tree and the species tree are displayed.

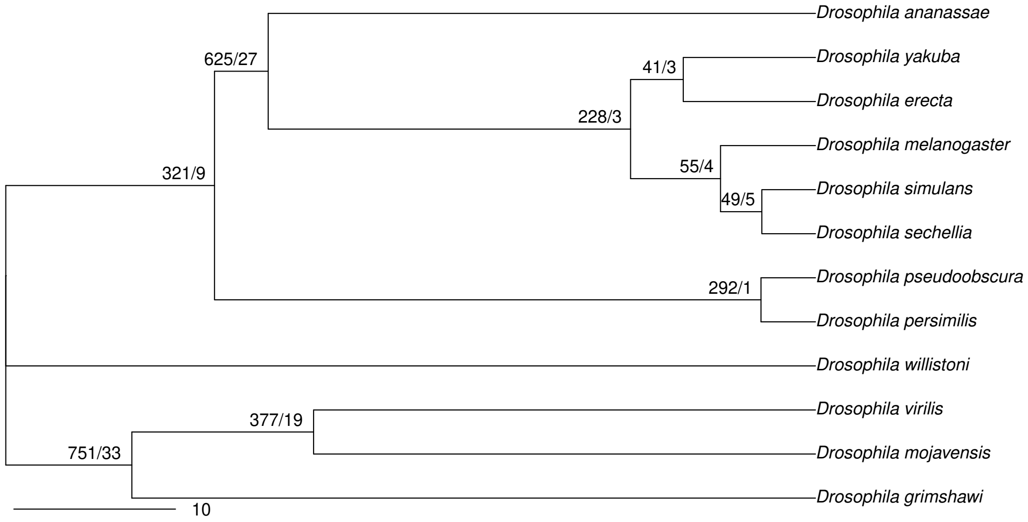

Figure S13: Phylogenetic tree of Drosophila species used in the study. Tree topology and branch lengths in millions of years were acquired from TimeTree (`http://www.timetree.org/`). Labels indicate the number of genes identified as evolving under positive selection at a particular branch (FDR threshold 0.1). The first numbers correspond to the branch-site model without rate variation, the second numbers correspond to the branch-site model with codon gamma rate variation. Only tests performed on branches compatible between the gene tree and the reference tree are displayed.

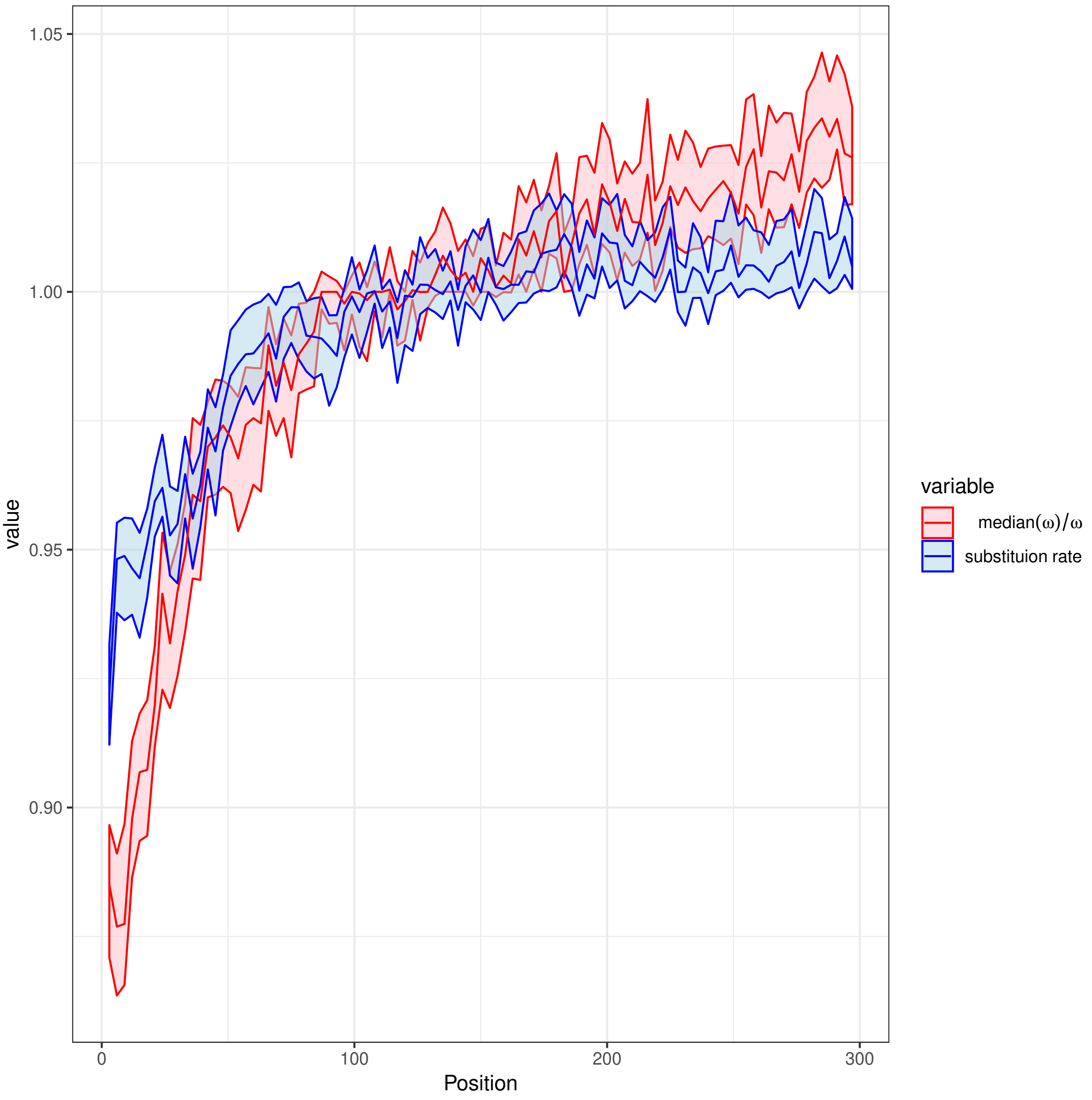

Figure S14: Median posterior estimates of nucleotide substitution rate and inverse protein substitution rates. The latter are defined as $\frac{median(\omega)}{\omega}$; median in the nominator is computed per gene alignment. Displayed as a function of distance from the start codon expressed in the number of nucleotides in Drosophila. The model M8 with codon gamma rate variation was used to estimate both parameters simultaneously. A value of 1 corresponds to the average (nucleotide substitutions) or median (protein substitutions) rate. The ribbons indicate 98% confidence intervals of median estimates. Start codons and alignment positions with less than three sequences were excluded from the plot.

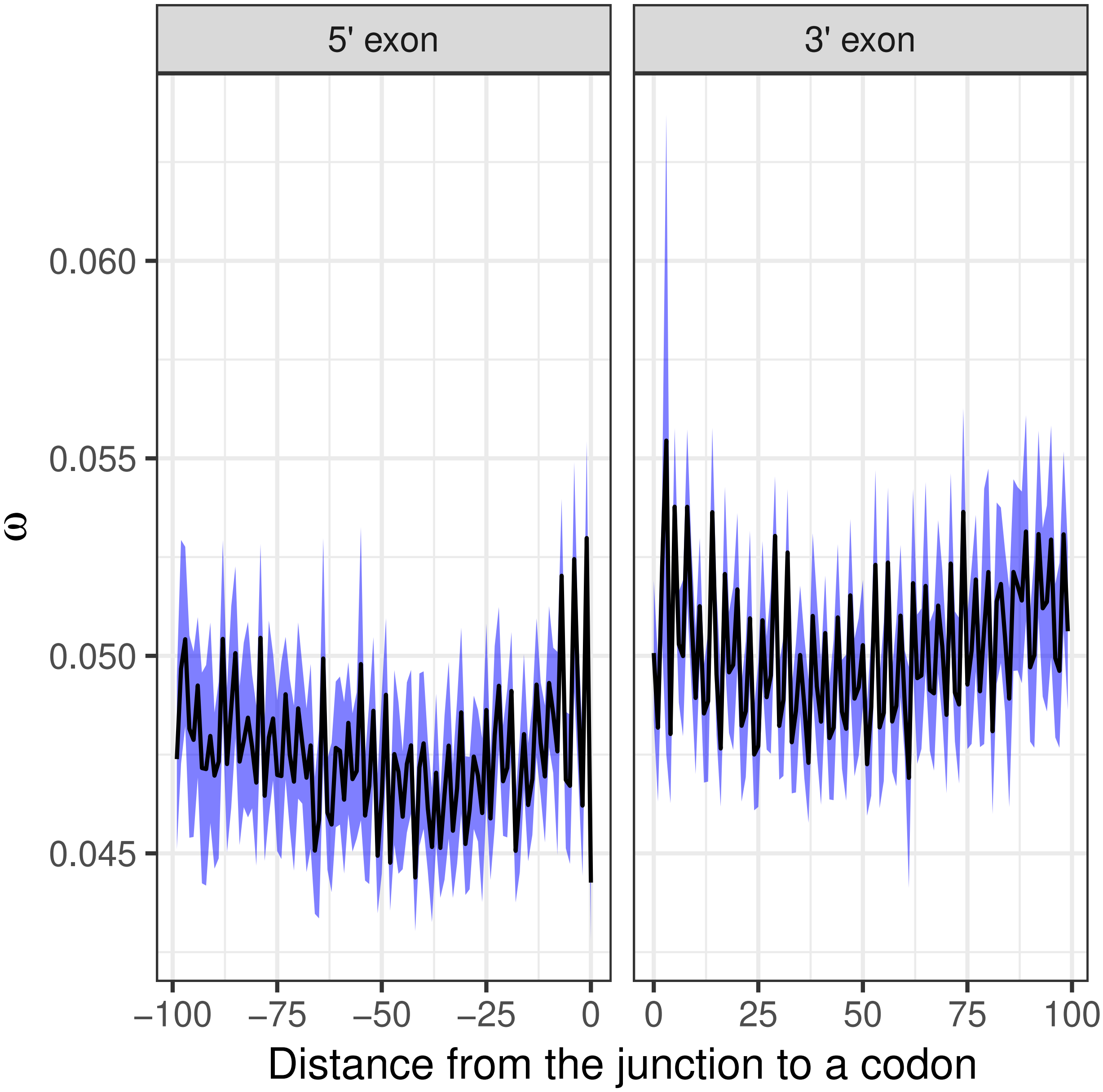

Figure S15: Selection parameter ω as a function of proximity to the exon-intron and intron-exon junction in the Drosophila dataset. The left panel depicts rates in 5' exon (prior to the exon-intron junction, negative distances), while the right panel depicts 3' exon (rates after the intron-exon junction, positive distances). The blue ribbon indicates 98% confidence interval of mean estimate. Only alignment positions with less than 30% of gaps were used in the plot.

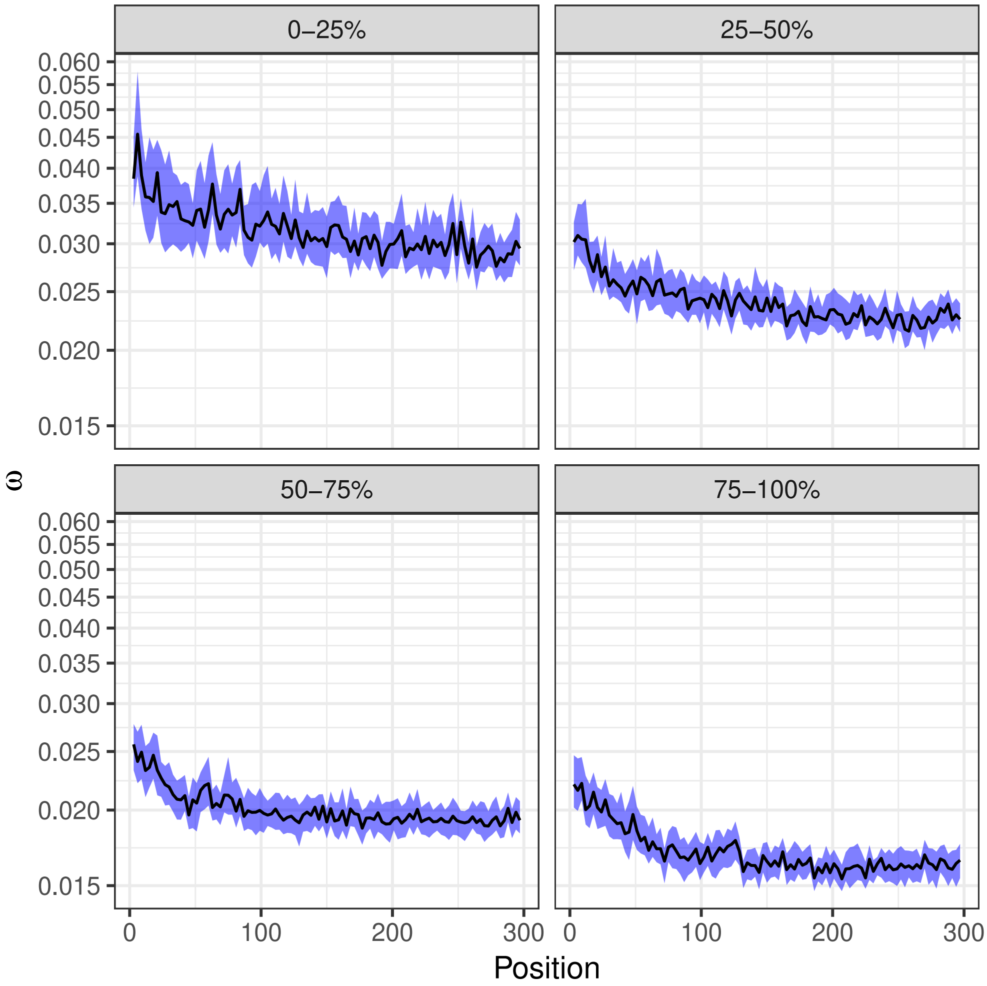

Figure S16: Posterior estimates of median ω as a function of distance from the start codon expressed in the number of nucleotides. The plot is split into four panels according to mean gene expression quantiles ($[0, 0.25)$, $[0.25, 0.5)$, $[0.5, 0.75)$ and $[0.75, 1]$, percentiles indicated on top of the panels). Logarithmic scale is used for the y axis. The M8 with codon rate variation model was used to estimate codon position specific ω. Smaller values of ω indicate stronger negative selection acting on the protein sequence. The blue ribbons indicate 98% confidence interval of median estimate. Start codons and alignment positions with less than three sequences were excluded from the plot.

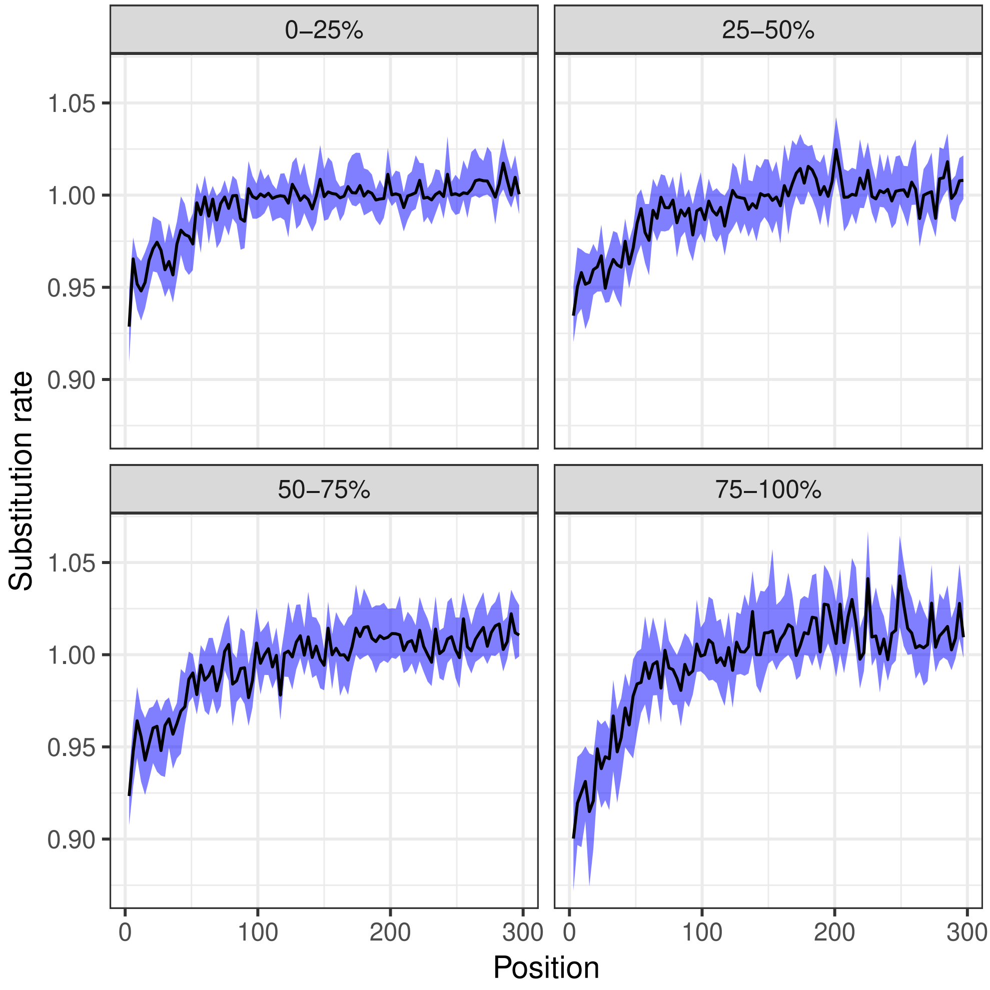

Figure S17: Posterior estimates of median codon substitution rate as a function of distance from the start codon expressed in the number of nucleotides. The plot is split into four panels according to mean gene expression quantiles ($[0, 0.25)$, $[0.25, 0.5)$, $[0.5, 0.75)$ and $[0.75, 1]$, percentiles indicated on top of the panels). The M8 with codon rate variation model was used to estimate the codon position rate. A substitution rate of 1 corresponds to the average rate of substitution over the gene; thus values above 1 do not indicate positive selection, but simply a rate higher than average for this gene. The blue ribbons indicate 98% confidence interval of median estimate. Start codons and alignment positions with less than three sequences were excluded from the plot.

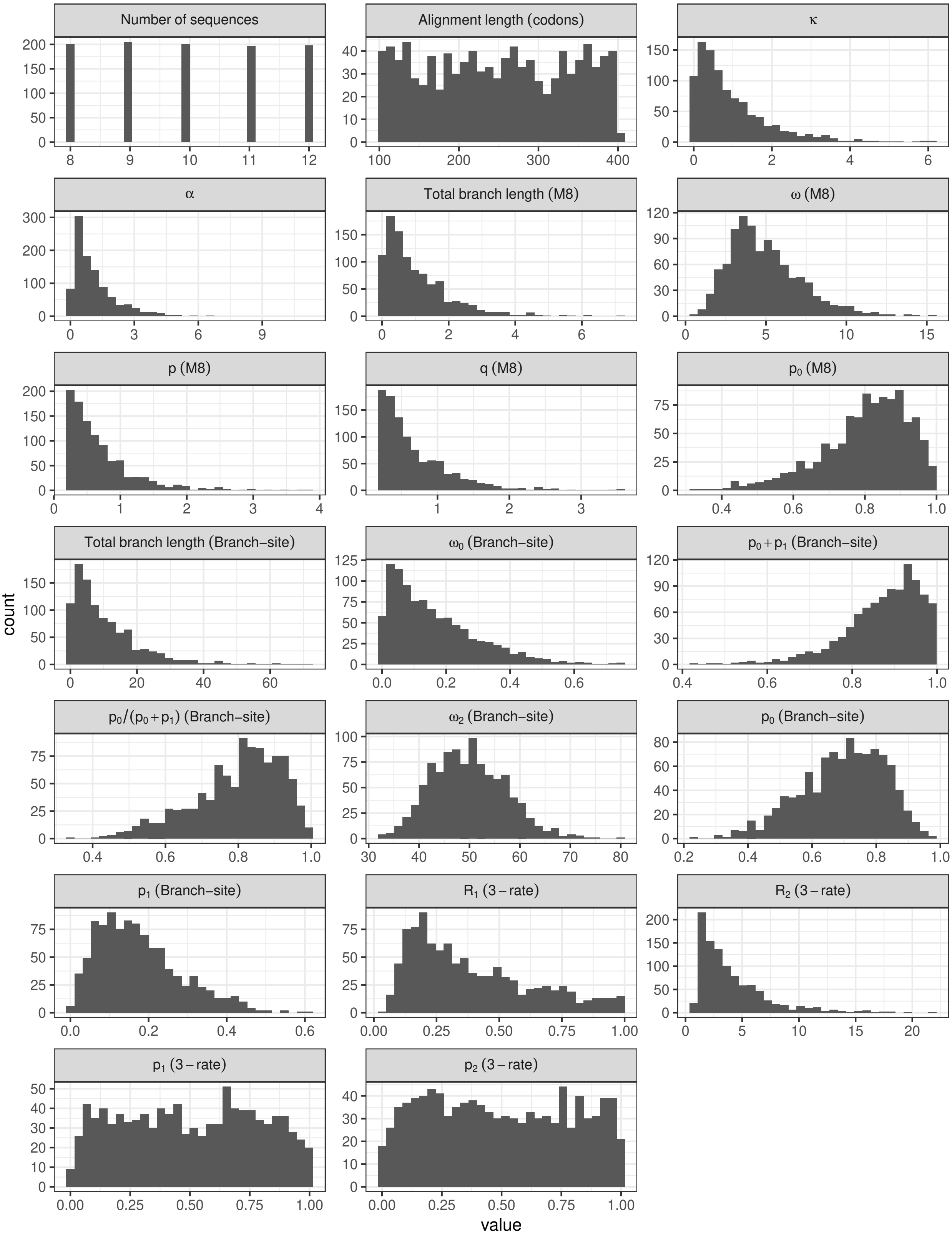

Figure S18: Distribution of simulation parameters.

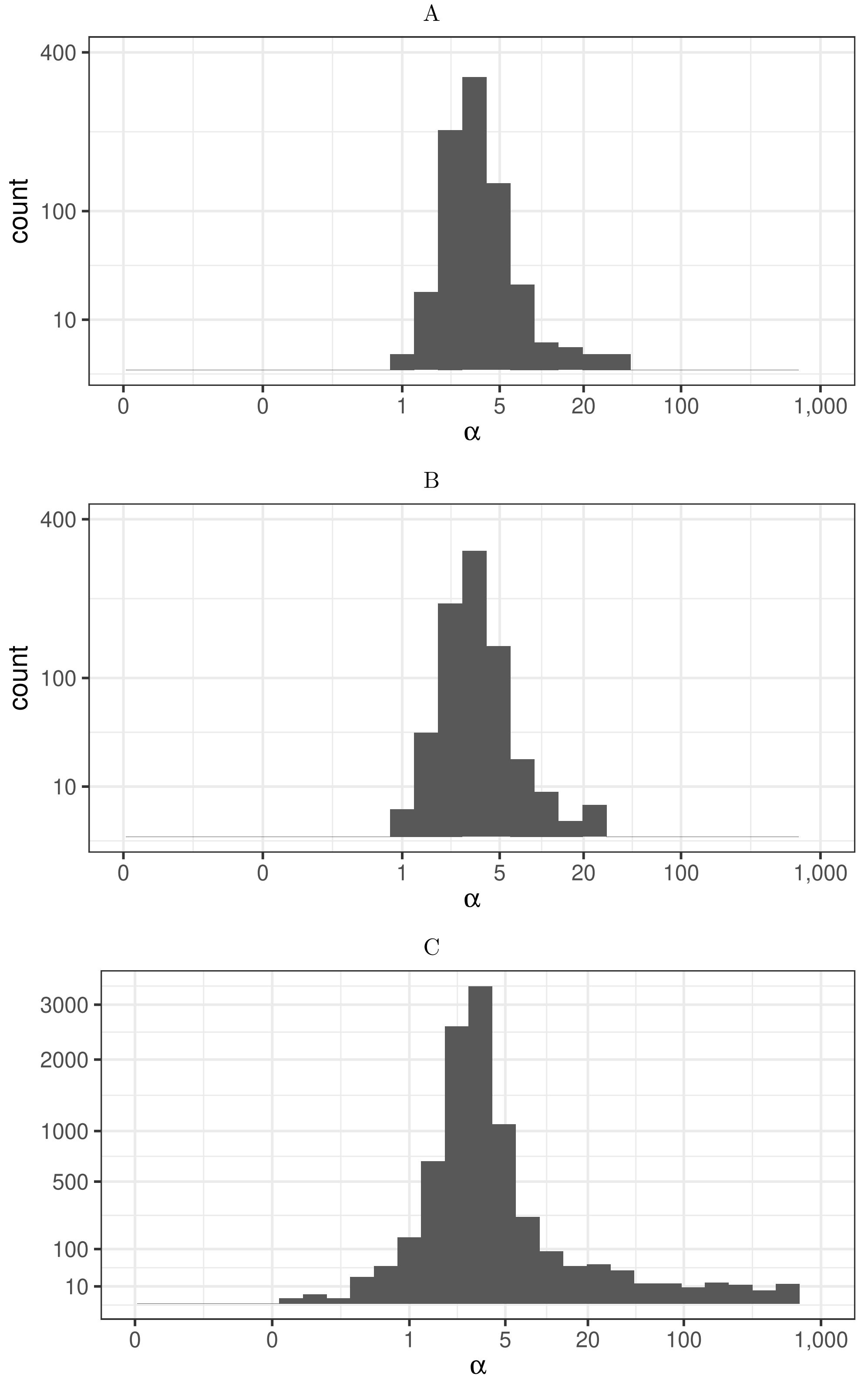

Figure S19: Estimates of α for: A) The vertebrate dataset, codon rate variation; B) the vertebrate dataset, site rate variation; C) the Drosophila dataset. The x axis is log-scaled, the y axis is scaled using the square root.

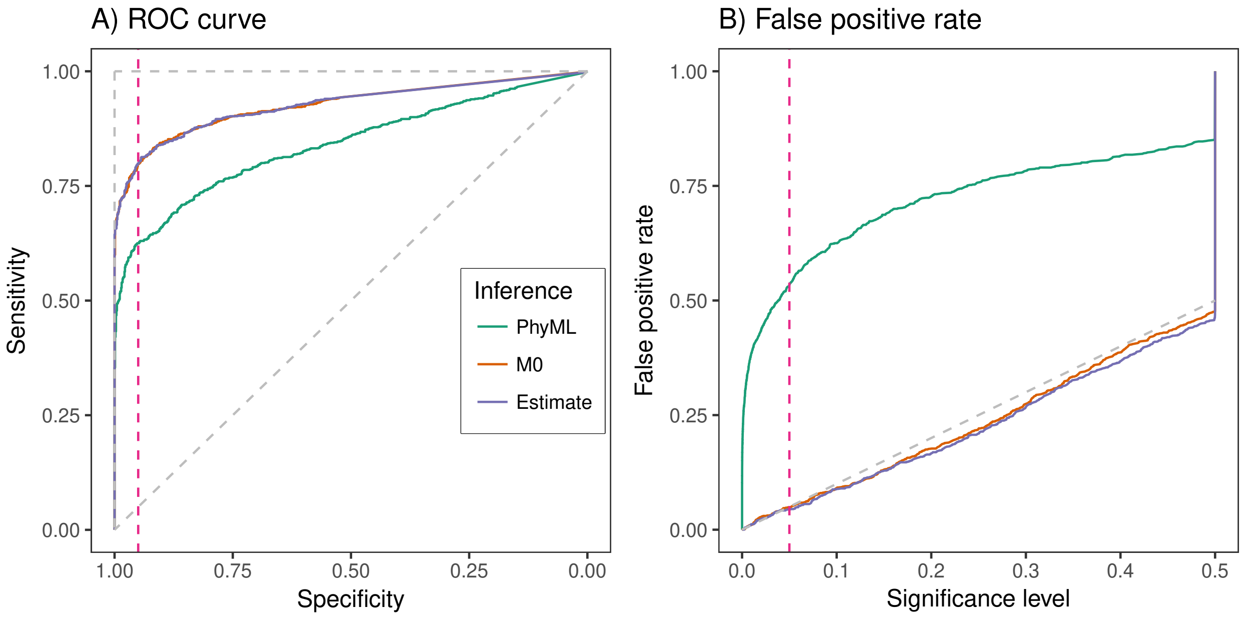

Figure S20: Effect of branch length estimation on positive selection prediction for the M8 model (no rate variation), data simulated under the same model. Three approaches were used to branch length estimation: keep PhyML estimates, estimate using M0, estimate branch lengths and model parameters simultaneously (see legend). A) ROC curve. B) False positive rate as function of significance level. The diagonal dashed line corresponds to the A) performance of a random predictor B) expected false positive rate under the null hypothesis. The vertical pink line corresponds to A) specificity of 95% (false positive rate of 5%), B) significance level of 5%.

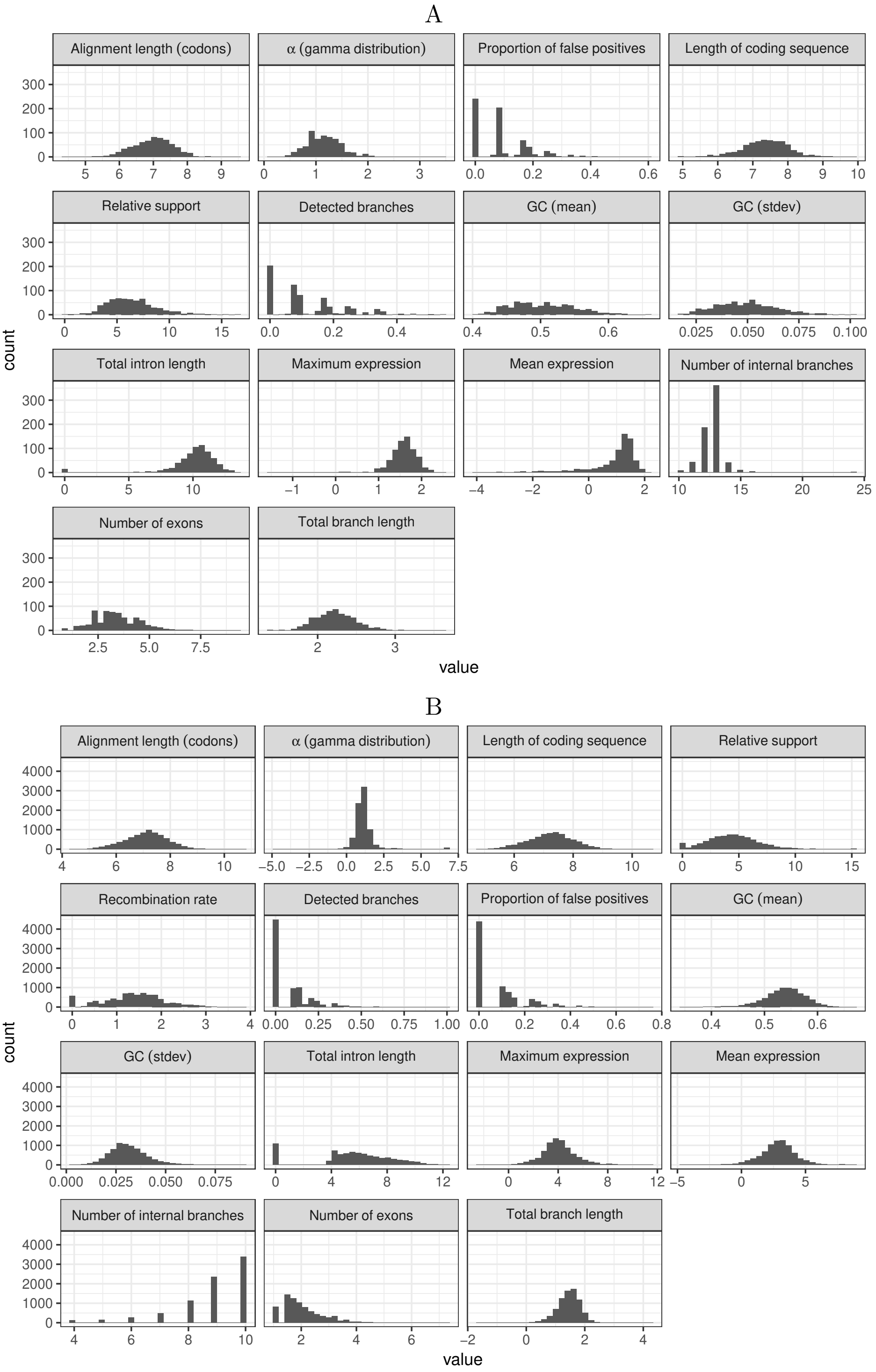

Figure S21: Distribution of transformed variables for the linear model. A) Vertebrate dataset, B) Drosophila dataset. Detected branches is proportion of branches detected using the branch-site model with codon gamma rate variation; proportion of false positives is proportions of branches which are detected by the model without rate variation but not detected by the model with codon gamma rate variation.

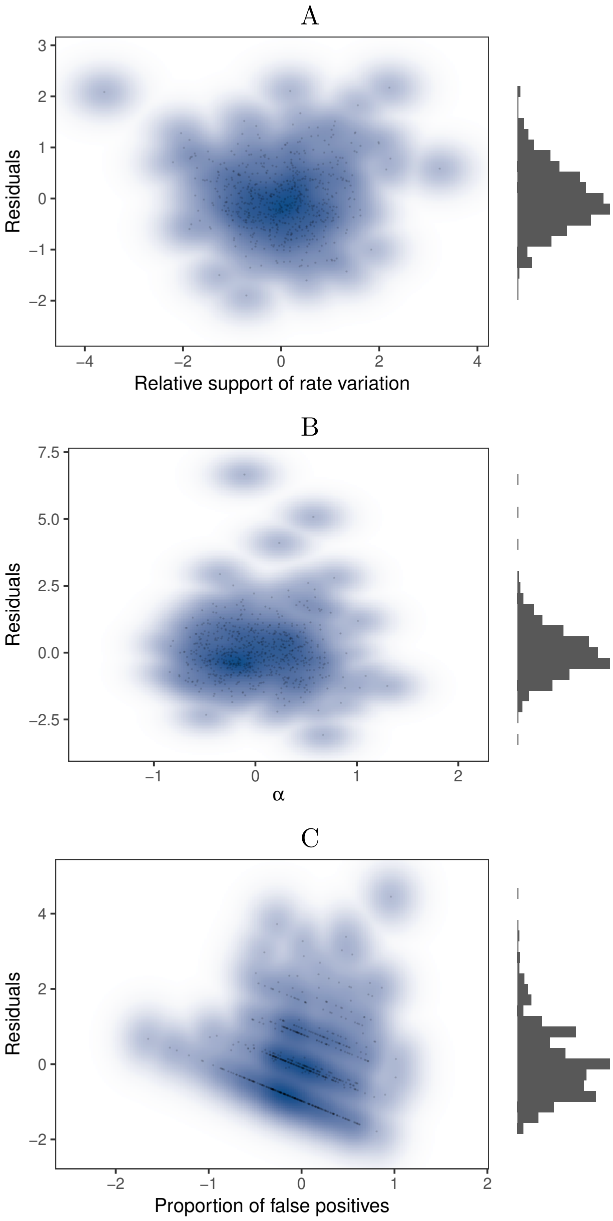

Figure S22: Linear model residuals as a function of the response variable for the vertebrate dataset: A) Relative codon rate variation model support, B) α parameter of gamma distribution, C) Proportion of branches detected by the model without rate variation but not detected by the model with codon gamma rate variation. Response variables were transformed (see methods). Residual histograms are depicted on the right. Points density is indicated with the blue background.

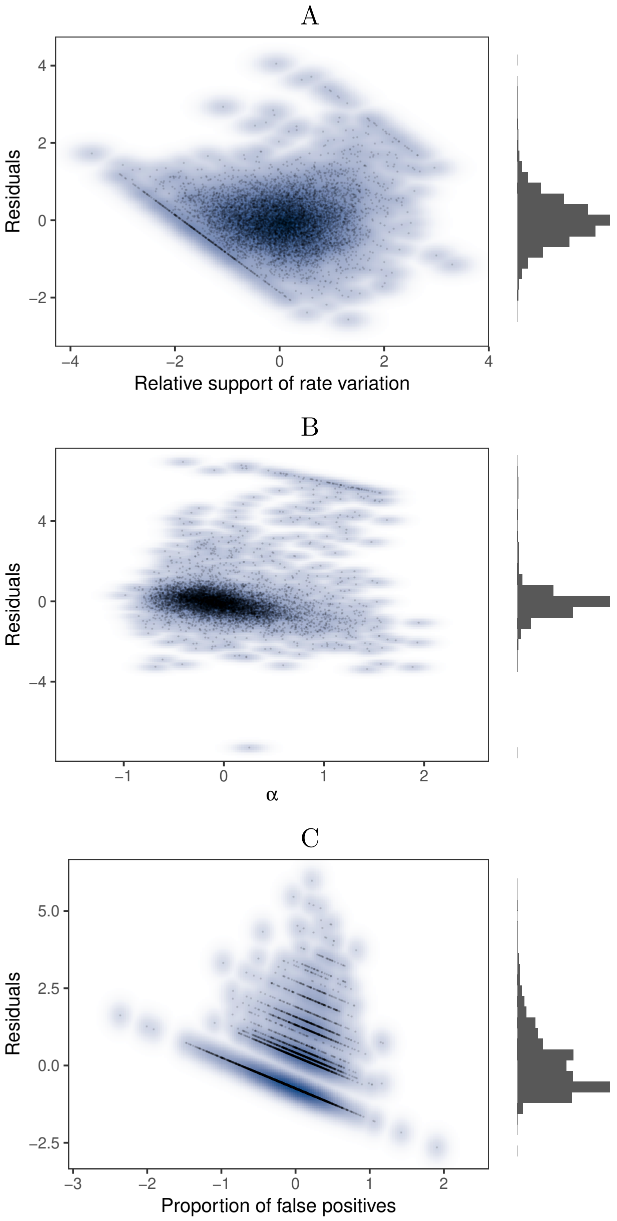

Figure S23: Linear model residuals as a function of the response variable for the Drosophila dataset: A) Relative codon rate variation model support, B) α parameter of gamma distribution, C) Proportion of branches detected by the model without rate variation but not detected by the model with codon gamma rate variation. Response variables were transformed (see methods). Residual histograms are depicted on the right. Density is indicated with the blue background.
